# Chorionicity and Heritability Estimates from Twin Studies: The Prenatal Environment of Twins and Their Resemblance Across a Large Number of Traits

**DOI:** 10.1007/s10519-015-9745-3

**Published:** 2015-09-26

**Authors:** C. E. M. van Beijsterveldt, L. I. H. Overbeek, L. Rozendaal, M. T. B. McMaster, T. J. Glasner, M. Bartels, J. M. Vink, N. G. Martin, C. V. Dolan, D. I. Boomsma

**Affiliations:** Department of Biological Psychology, VU University Amsterdam, Van der Boechorststraat 1, 1081 BT Amsterdam, Netherlands; PALGA Foundation, Utrecht, Netherlands; Genetic Epidemiology Unit, Queensland Institute of Medical Research, Brisbane, QLD Australia; Department of Pathology, VU University Medical Centre, Amsterdam, Netherlands

**Keywords:** Chorion, Record linkage, Equal environment, Prenatal, Twins, Heritability estimate

## Abstract

There are three types of monozygotic (MZ) twins. MZ twins can either share one chorion and one amnion, each twin can have its own amnion, or MZ twins can—like dizygotic twins—each have their own chorion and amnion. Sharing the same chorion may create a more similar/dissimilar prenatal environment and bias heritability estimates, but most twin studies do not distinguish between these three types of MZ twin pairs. The aim of this paper is to investigate the effect of chorion sharing on the similarity within MZ twin pairs for a large number of traits. Information on chorion status was obtained for the Netherlands twin register (NTR) by linkage to the records from the database of the dutch pathological anatomy national automated archive (PALGA). Record linkage was successful for over 9000 pairs. Effect of chorion type was tested by comparing the within-pair similarity between monochorionic (MC) and dichorionic (DC) MZ twins on 66 traits including weight, height, motor milestones, child problem behaviors, cognitive function, wellbeing and personality. For only 10 traits, within-pair similarity differed between MCMZ and DCMZ pairs. For traits influenced by birth weight (e.g. weight and height in young children) we expected that MC twins would be more discordant. This was found for 5 out of 13 measures. When looking at traits where blood supply is important, we saw MCMZ twins to be more concordant than DCMZ’s for 3 traits. We conclude that the influence on the MZ twin correlation of the intra-uterine prenatal environment, as measured by sharing a chorion type, is small and limited to a few phenotypes. This implies that the assumption of equal prenatal environment of mono- and DC MZ twins, which characterizes the classical twin design, is largely tenable.

**Electronic supplementary material:**

The online version of this article (doi:10.1007/s10519-015-9745-3) contains supplementary material, which is available to authorized users.

## Introduction

The classical twin design has been the most productive method to assess heritability in the analysis of complex human traits. Its widespread application has established the importance of genetic effects on a very large number of complex traits in many populations. Notwithstanding its utility, this design is based on several assumptions, and this has raised valid questions concerning the results that it has produced. One source of possible bias in twin-based heritability estimates is chorion sharing, i.e., an aspect of the prenatal environment, which differs in monozygotic (MZ) and dizygotic (DZ) twins (Phelps et al. 1997; Phillips 1993; Price 1950).

Monochorionic (MC) MZ twins (MCMZs; ~66 % of MZ twin pairs) are thought to originate in late cleavage division of the blastocyst. MCMZs share a single placenta, and so all blood-borne factors, emanating from the mother or the placenta. Monochorionicity may be associated with vascular anastomosis (twin-to-twin blood transfusion), which is disadvantageous to both twins. MCMZ twins can either share one amnion, or each have their own amnion [i.e. they may be monoamnionitic (MA) or diamniotic (DA)]. DCMZs are thought to arise earlier during the morula stage, and, like DZ twins, have individual placentas.

Any bias in twin based heritability estimates attributable to MZ chorionicity will depend on the direction and magnitude of its effect (Prescott et al. 1999). An increase in MCMZ resemblance may result in an overestimation of (broad-sense) heritability, although, remarkably, earlier publications were mainly concerned about underestimation of heritability because of MCMZs For example, Price (1950) wrote “In all probability the net effect of most twin studies has been underestimation of the significance of heredity in the medical and behavior sciences”.

The question of bias is important in its own right, but has recently come to the fore, in the discussion on the missing heritability. This concerns the discrepancy between the magnitude of twin-based heritability estimates and the, appreciably lower, amount of genetic variance explained by polygenic scores in genome-wide association studies (Walter et al. 2013) and by chip-based heritability studies (Yang et al. 2010; Zaitlen et al. 2013). Many explanations have been advanced to account for this discrepancy (Eichler et al. 2010; Manolio et al. 2009), including possible overestimation of heritabilities based on the twin design (Zuk et al. 2012).

Studies of the effects of chorionicity on a wide range of physical and psychological phenotypes have produced mixed results, with the possible exception of birth weight, for which a well-established result is the greater resemblance in birth weight of DCMZs versus MCMZs (Melnick et al. 1978; Sokol et al. 1995; Gutknecht et al. 1999; Corey et al. 1976; Fagard et al. 2003; Hur 2007; Hur and Shin 2008; Jacobs et al. 2001; Loos et al. 2001a, 2005; Race et al. 2006; Reed et al. 2002; Rose et al. 1981; Vlietinck et al. 1989). Many studies are characterized by small sample sizes, and heterogeneity with respect to age of the twins. Here we present results of the analysis of 66 phenotypes, observed in relatively large samples of MZ twins, who were followed from birth into young adulthood. We consider height and weight, attainment of motor development milestones, infant temperament, personality measures, dimensions of psychopathology, and measures of cognitive ability.

## Methods

### Sample

**Fig. 1:**
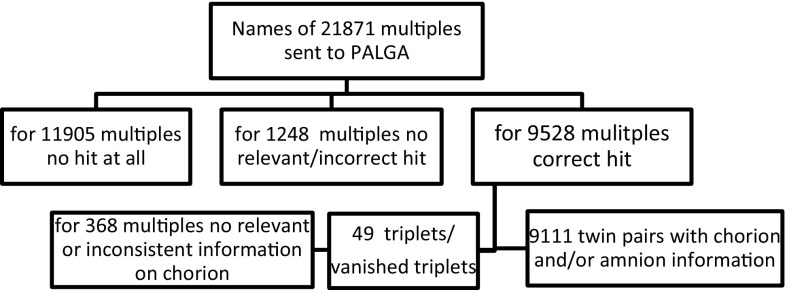
Overview of the result of the linkage between records of the NTR and PALGA Database

The population based Netherlands twin register (NTR: www.tweelingenregister.org) has recruited newborn twins in the Netherlands since 1989 (Glasner et al. 2013). About 40 % of all newborn twins (and higher order multiples) are registered by their parents. Parents and teachers participate in longitudinal survey studies and provide information on the development of the children in the first 12 years of life. After age 12, twins and their siblings provide self-ratings. Outcome variables concern health, cognition, behavioural, and emotional problems. The sample is a representation of twin families in the general population (Boomsma et al. 2006; van Beijsterveldt et al. 2013). In 2005, permission for record linkage was obtained from all mothers, who were registered with the NTR at that time (Hoekstra et al. 2008). From 2005 onwards, the permission to link NTR data to other databases in the Netherlands is asked of parents of twins at time of registration with the NTR. Over 90 % of the mothers granted this permission. For multiples born between 1986 and 2011, we ascertained permission for record linkage from 21 698 mothers, including 173 mothers with two sets of twins (thus for 21,871 multiples). The NTR records of these participants were linked to the Pathological Anatomy National Automated Archive (PALGA: http://www.palga.nl) to retrieve information on chorion type. PALGA started to store abstracts of pathology reports in 1971, and since 1991 all Dutch pathology laboratories have been linked (Casparie et al. 2007). An abstract consists of encrypted patient identification, a summary of the report and diagnostic terms in line with SNOMED terminology (systematized nomenclature of medicine clinical terms). About 64 million abstracts pertaining to over 12 million patients are stored. Information about placenta and chorion type of multiples born in Dutch hospitals is included in this database. To obtain an estimate of the percentage of placentas of multiples included in the PALGA database, we compared the number of patients with a pathology report on a multiple birth with the number of twin deliveries in the Netherlands (from the Database of Statistics Netherlands (CBS 2013)). For the period 1991–2011, a rough estimate of the coverage of multiple placental data is ~46 %, but there has been a gradual decrease of the percentage from ~60 % in 1991–33 % in 2011 (see Table S1 Online Supplementary Materials I). To link the records of NTR mothers to the records of the PALGA database, we used the following patient identifiers: mother’s date of birth (DOB), sex, the first four characters of the mother’s maiden name, date of receipt of the biological sample(s), and postal code as optional identifier. DOB of the twins and triplets (±7 days) is used as proxy for the date of receipt (±7 days) in the PALGA records. Person identifiers were changed into pseudonyms by a Trusted Third Party (ZorgTTP, Houten). Figure 1 gives a systematic overview of the record linkage results. For 50 % of NTR participants we obtained no hit at all, and in 6 % we obtained a hit, but the data turned out to be irrelevant or unreliable. Record linkage was successful for 44 % of the multiples, closely corresponding to the 46 % estimated coverage of the PALGA database. Table S2 (Online Supplementary Materials I) contains maternal and birth characteristics of the group with and without successful record linkage (misclassifications were not included). Although most multiple deliveries occurred in a hospital (The Netherlands Perinatal Registry, Utrecht, The Netherlands), only a selected sample of multiple placentas was sent to the pathology laboratory. Compared to the twins without successful linking, the twins in the linked PALGA group were more often MZ, had lower birth weights, had higher rates of birth weight discordance, had shorter gestational ages, were delivered more often by C-section, and were more often spontaneously conceived. The mothers in the linked PALGA group were younger, and tended to live in urban areas. However, the effect size of urban area was very small [effect size (Cramer’s v) = 0.03].

### Chorionicity, zygosity and types of twins

Information on chorionicity in linked records was retrieved from PALGA text files that contained the summary (conclusion) of the pathology report. The pathology reports provided relevant information on chorion and/or amnion in 9111 MZ and DZ pairs. In 8982 cases, information was available on both chorion and amnion, in 89 cases information was limited to chorion, and in 40 cases, information was limited to amnion.

In the successfully linked cases, zygosity information obtained from the NTR was linked to chorionicity information (for 131 pairs there was no information on zygosity; for 27 MZ pairs, zygosity was known on the basis of the chorionicity, leaving 104 DC twin pairs with unknown zygosity). Table 1 gives an overview of the classification of twin pairs and zygosity.

**Table 1:** Classification of twin pairs according chorionicity and amnionicity

	Chorion		Amnion		
	DC	MC	MC		
Zygosity			MA	DA	Missing
MZ males	595	1182	80	1058	44
DZ males	1615	(108)	0	0	
MZ females	647	1288	127	1122	39
DZ females	1515	(98)	0	0	
DZ MF	958	(31)	0	0	
DZ FM	903	(27)	0	0	

The number within the brackets represents the falsely classified cases

*MC* monochorionic, *MA* monoamniotic, *DC* dichorionic, *DA* diamniotic, *MZ* monozygotic, *DZ* dizygotic

Zygosity of the same-sex twins was determined as follows: 11 % by blood/DNA polymorphisms, 65 % by parental report on items about physical similarity and frequency of confusion of the twins by parents and strangers [against blood/DNA polymorphisms this classification is correct for 93 % (Rietveld et al. 2000)], 10 % by a single item that indicates how much the children look alike [against blood/DNA polymorphisms this classification is correct for 92 % (Groen-Blokhuis et al. 2011)], and the remaining 14 % of the twins by the maternal answer on the question whether the zygosity of the twin pair was MZ, DZ, or unknown. Based on the shared number of chorions and amnions, twin pairs were classified as MC or DC and mono- amnionitic (MA) and diamniotic (DA). Because DZ twin pairs are always DC (and thus DA), highly unlikely combinations, i.e. MCDZ were excluded from the analyses (n = 265). It should be noted that there are reports of DZ twins who share a chorion, but such cases are extremely rare (Souter et al. 2003; Miura and Niikawa 2005). Consistent with the percentage given by Hall (2003), 33.5 % of the MZ twin pairs were classified as DCMZ. MA is a rare phenomenon, among all MZ pairs, 5.7 % were MA. We acknowledge that 5.7 % is higher than the percentage reported by Hall (2003; 1–2 %) and Derom et al. (1988; 1.64 %), and by Machin and Still (1995; 2.3 %). However, our 5.7 % is more comparable to the 4 % reported by the Australian twin registry (http://www.twins.org.au/).

### Phenotypes

*Growth* After registration of their twins, mothers reported birth weight and height in a survey sent within the first year after birth (Gielen et al. 2010). Birth weight discordance (BWD) was calculated as follows: [(largest birth weight -smallest birth weight)/largest birth weight]*100. Weight and height between birth date and age three were obtained by mother report of the data retrieved from the routine health care program in the Netherlands (Youth Health Services). In the survey collected at age 5, a parent was asked to report weights and heights from age 3–5. In the follow-up surveys at ages 7, 10, 12 years, parents reported current weight and height. From age 14 onwards, weight and height were self-reported by the twins.

*Motor milestones* When the twins were 2 years old, the mother was asked to report the age at which certain motor milestones were reached: turning over from back to belly (turning), sitting without support (sitting), crawling on hands and knees (crawling), standing without support (standing) and walking without support (walking) (Brouwer et al. 2006; Langendonk et al. 2007).

*Early temperament* In a subsample of the survey at age 2, the Emotionality, Activity, and Sociability (EAS) Temperament Survey (Mathiesen and Tambs 1999) was collected (from 2008). The EAS consists of 20 items with a 5-point scale response format, and measures four phenotypes (five items each): shyness, activity, emotionality, and sociability.

*Asthma and eczema:* In the survey of 5-year olds, a parent was asked to report (yes/no) whether a physician had ever diagnosed the following conditions: asthma, eczema, bronchitis, and pneumonia (van Beijsterveldt and Boomsma 2007).

*Handedness* Handedness (left-, right-handed or both) was obtained by self-report and parental report about which hand is used for drawing at the survey of age 5 (Medland et al. 2009).

*Behavior and emotional problems* Maternal and self-report ratings of behavioral and emotional problems were collected by using age appropriate versions of the Achenbach System of Empirical Based Assessment (ASEBA) (Achenbach and Rescorla 2013) and Conners’ Parent Rating Scale-Revised (CPRS-R) (Conners et al. 1998). Details of data collection are described elsewhere (Bartels et al. 2004; Derks et al. 2004). From the ASEBA surveys, we used the Externalizing, Internalizing, and Autism scale (So et al. 2013). This study uses Dutch syndrome scales (Koot et al. 1997), which are comparable with the syndrome scales as developed by (Achenbach 1992). From the CPRS-R, the ADHD-index was used, consisting of 12 items.

*Cognitive performance* At age 12, we collected the ‘CITO-scores’, a score on the standardized national Dutch test for educational achievement, which is administered in the last grade of primary school. School performance of 10 year-old children was assessed by teacher ratings on a 5-point scale from one (insufficient) to five (very good), for the domains, arithmetic, language, reading, and gymnastics (de Zeeuw et al. 2012).

*Personality* neuroticism, extraversion, openness to experience, agreeableness and conscientiousness were assessed by the NEO-FFI (Costa and McCrae 1992; Hoekstra et al. 1996). Personality data were obtained by self-report from the age of 16 years and older (Willemsen et al. 2013).

*Subjective well-being (SWB)* SWB was assessed by self-report in adolescents. A SWB factor score was constructed from the measures of subjective happiness, satisfaction with life, and quality of life (Bartels et al. 2013).

### Statistical analyses

As a first step we examined the representativeness of the successfully linked group. Birth and maternal characteristics were compared between the group with and without successful record linkage (misclassifications were not included). Categorical variables were tested by Chi squared tests. Continuous variables were tested in ANOVAs and linear mixed models (SPSS 2012).

To test the effect of chorion type, we compared the trait resemblance of the MCMZ and DCMZ pairs. Because MZs are genetically identical, differences in resemblance between MCMZ and DCMZ pairs are due to the environment, which includes the prenatal environment. To examine the MCMZ versus DCMZ differences in resemblance in continuous phenotypes, we used likelihood-ratio tests of the means, the variances, and the intra-class correlation (icc). In the analysis of binary phenotypes, we used the liability threshold model (Neale and Cardon 1999), and used likelihood-ratio tests to examine threshold differences and differences in intra-class correlations at the level of the liability. These analyses were carried out in OpenMx in the R programs (Boker et al. 2011, 2012). We carried out a sensitivity analysis to gauge the effect of zygosity and chorionicity misclassification on the twin correlations in the MCMZ and DCMZ groups. The results of these analyses are included in Supplementary Material II and summarized under “limitations” in the Discussion of the paper.

## Results

Table 2 displays the *maternal and birth characteristics* for MC, monoamniotic (MA), DC, and diamniotic (DA) MZ and DZ same-sex twin pairs. In testing for mean differences between the types of twins we applied a significance level of 0.0055 (0.05/9, Bonferroni correction for 9 variables). There was an overall effect on gestational age (F = 4.874, df = 3; p = 0.002), and post hoc comparisons revealed significant differences between DZ and MCMA pairs and between DZ and DCMZs, with DZ pairs having the longest gestational age. Age of mother was significantly different between the 4 groups (F = 29.527; df = 3; p < 0.001). DZ mothers were significantly older than mothers of MCMA and MCDA MZ pairs. The mothers of the DCMZ pairs were significantly younger than the MCDA pairs. The means of percentage BWD differed significantly between the 4 types of pairs (F = 15.275; df = 3; p < 0.001). The highest BWD was observed in the DZ pairs, which differed significantly from DCMZ pairs. In addition, the BWD of MCDA MZs was significantly larger than the BWD of DCMZs. There was a significant overall effect of birth weight for first-born twins (F = 9.292; df = 3; p < 0.001), but not for second-born twins (F = 3.681; df = 3; p = 0.012). DZ first-born twins were heavier than the other three types of MZ pairs. In the second-born twins, birth weight was only different between DZ and MCDA MZ twins. There was no effect of twin type on birth height (first-born: F = 2.826; df = 3; p = 0.037; second-born: F = 2.350; df = 3; p = 0.07) and number of days in incubator (first-born: F = 3.808; df = 3; p = 0.01; second-born: F = 1.095; df = 3; p = 0.35).

**Table 2:** Maternal and birth characteristics by type of same-sex twin pairs

	Type of twin											
	MCMA MZ			MCDA MZ			DCMZ			DC DZ		
	N	Mean	SD	N	Mean	SD	N	Mean	SD	N	Mean	SD
Gestational age^a^	196	35.7	(2.9)	2083	36.0	(2.6)	1208	36.0	(2.8)	2935	36.2	(2.8)
Maternal age at birth^a^	207	31.1	(3.9)	2180	31.0	(4.1)	1242	30.6	(3.9)	3130	31.7	(3.8)
% Birth weight discordance^a^	196	11.6	(9.3)	2075	12.2	(9.9)	1205	10.4	(9.2)	2913	12.7	(10.3)
Birth height first born	148	45.7	(3.8)	1504	45.9	(3.9)	856	45.9	(3.9)	2093	46.2	(4.0)
Birth height second born	147	45.6	(3.6)	1500	45.7	(3.9)	860	45.9	(3.9)	2091	46.0	(4.0)
Birth weight first born^a^	196	2336.4	(558.6)	2070	2387.3	(547.5)	1203	2382.8	(565.0)	2909	2456.3	(584.3)
Birth weight second born	196	2308.8	(580.8)	2074	2331.1	(564.1)	1204	2349.4	(563.3)	2906	2381.4	(585.4)
Days in incubator first born	184	8.1	(12.6)	1987	6.2	(11.9)	1167	6.6	(12.6)	2771	5.6	(12.1)
Days in incubator second born	183	7.8	(12.1)	1981	6.9	(12.8)	1160	7.2	(12.9)	2752	6.6	(13.0)

*MC* monochorionic, *MA* monoamniotic, *DC* dichorionic, *DA* diamniotic, *MZ* is monozygotic, *DZ* dizygotic

^a^Indicates an overall significant effect (α = 0.0055)

Table 3 displays maternal and birth characteristics for the categorical measures. The significance level was determined at 0.0074 (0.05/7 Bonferroni correction for 7 discrete variables). For Caesarian section (C-section) (Wald = 12.247; df = 1; p < 0.001) and IVF/ICSI (Wald = 173.566; df = 1; p < 0.001), a significant effect of zygosity was found. Deliveries by C-sections and IVF/ICSI were more common for DZ than for MZ twins. As the number of MZ twins born after IVF is small and we have previously detailed that for a large number of traits, the development in IVF and naturally conceived twins is similar (Van Beijsterveldt et al. 2011) we do not consider the role of IVF any further. Health problems shortly after birth of first-born twins were more frequent in MC than in DC pairs (Wald = 9.435; df = 1; p = 0.002). There was a significant effect on %BWD of chorion (Wald = 21.739; df = 1; p < 0.001) and zygosity (Wald = 32.243; df = 1; p < 0.001). MCMZ pairs were more often discordant than DCMZ pairs with respect to birth weight. DZ pairs, because of their genetic differences, were most often discordant for birth weight. MZ pairs were more often born preterm than DZ pairs (t = 14.985; df = 1; p < 0.001).

**Table 3:** Maternal and birth characteristics (% yes) by type of same-sex twin pairs

					Chorion	Amnion	Zygosity
	MCMA MZ	MCDA MZ	DCMZ	DC DZ	p	p	p
IVF/ICSI	1.4 %	2.8 %	2.8 %	20.8 %	0.964	0.277	<0.001
C-section	33.1	28.5	28.4	34.5	0.952	0.196	<0.001
health problems shortly after birth, 1st born	30.2	29.9	24.6	25.4	0.002	0.933	0.629
health problems shortly after birth, 2nd born	36.2	32.7	33.8	32.7	0.540	0.322	0.534
% birth weight discordance >20%	18.4	19.4	13.0	20.6	<0.001	0.721	<0.001
% preterm (<37 weeks)	54.1	49.2	51.2	44.6	0.261	0.193	<0.001
% very preterm (<32 weeks)	8.2	6.6	8.4	7.5	0.065	0.413	0.344

*MC* monochorionic, *MA* monoamniotic, *DC* dichorionic, *DA* diamniotic, *MZ* monozygotic, *DZ* dizygotic

Table S3 (Online Supplementary Materials I) gives an overview of *congenital and chronic diseases* at age 3 years for the four types of twins. Here, the data from DZ pairs also include data from opposite-sex twin pairs. We could not apply the overall test to the very rare diseases (diabetes, cancer liver disease, chronic rheumatism, deafness, and blindness), because there are too few cases within cells. Of the remaining diseases, the effect of type twin was only significant on heart disease, with MCMA twins having the largest risk of heart disease (2.2 versus 0.3 % in the other groups of twins). Given the low numbers, a possible chance finding cannot be ruled out.

Next, we compared MCMZ and DCMZ pairs with respect to growth, motor milestones, temperament, cognition, personality, wellbeing, puberty stages, asthma and handedness. The means, standard deviations, and correlations of continuous traits for the MCMZ and DCMZ pairs are given in Table S4 (Online Supplementary Materials I). Given the number of comparisons (66 traits), we applied a Bonferroni correction on the significance level (alpha) to arrive at a family-wise alpha of 0.00075 (i.e., 0.05/66). For none of the traits significant mean differences between MCMZ and DCMZ twins were found. Variances differences were found for weight at age 7 and 12, and for internalizing problems at age 12, with higher variances in the MCMZ groups.

**Fig. 2:**
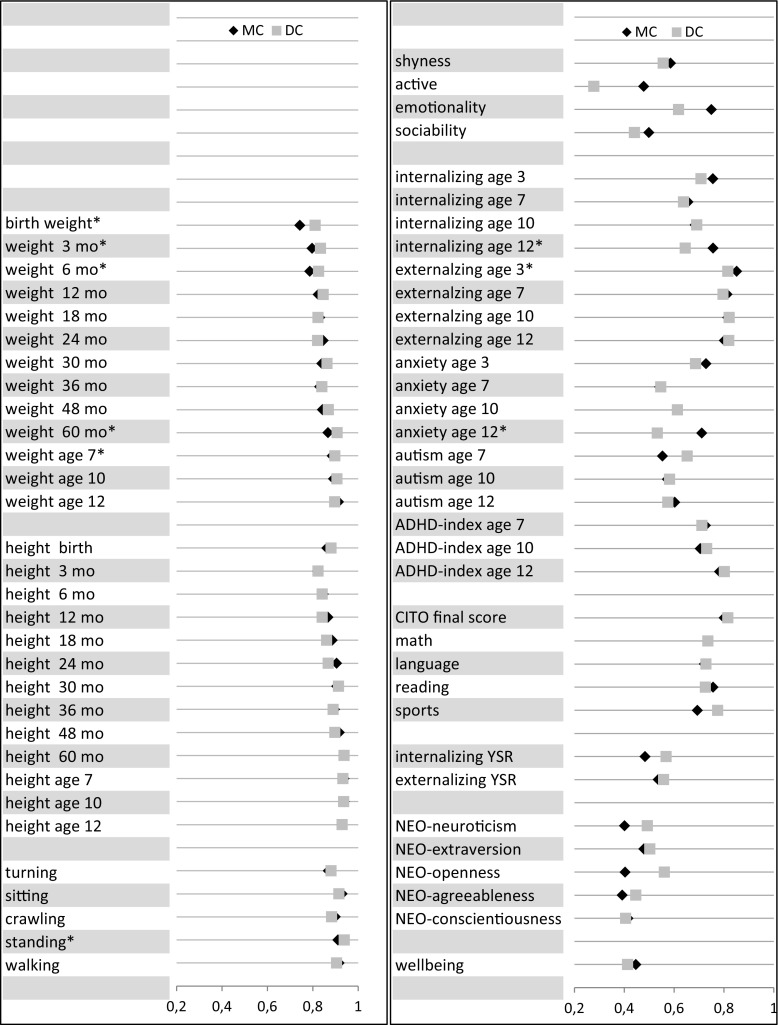
Overview of the intra-class correlations for MCMZ and DCMZ twin pairs (N MCMZ pairs varies between 2338 and 170 and N DCMZ pairs varies from 1203 to 66). *Asterisk* denotes a significant difference between the two groups

An overview of the MCMZ and DCMZ correlations is given in Fig. 2. For 56 out of 66 traits, the icc did not differ between MCMZ and DCMZ pairs. For weight, we found for 5 out of 13 measures that the correlations of MCMZs were lower than the DCMZs. The largest difference in correlations was found for birth weight (0.75 for MC versus 0.81 for DC) and smallest for weight at age 7 (0.89 for MCMZ versus 0.90 for DCMZ). For weight at 3, 6, and 60 months the differences in correlations were 0.04, 0.03, and 0.02, respectively. For the motor milestones standing, we found a significant difference in icc between MCMZ (r = 0.91) versus DCMZ (r = 0.94) pairs. For problem behaviors, MCMZ pairs were more similar for externalizing behavior at age 3 (r = 0.85 for MCMZ and r = 0.82 for DCMZ), for internalizing behavior at age 12 (r = 0.76 for MCMZ and r = 0.65 for DCMZ), and for anxiety at age 12 (r = 0.71 for MCMZ and r = 0.53 for DCMZ). For the autism scale differences were in reversed direction (r = 0.55 for MCMZ and r = 0.65 for DCMZ). For traits assessed in adolescents, there were no significant effects of chorion type. None of the correlations of the discrete variables differed between MCMZ and DCMZ pairs (Table S5 (Online Supplementary Materials I)).

## Power analyses

We considered the power to detect a difference in intra-class correlations given that the MCMZ and DCMZ groups did not differ in means or standard deviation. To obtain an indication of the magnitudes of the icc’s, we calculated the mean and standard deviation of the icc’s of the 66 continuous phenotypes in the two groups. The range of most icc’s was between 0.6 and 0.9, and the average icc was ~0.74 (SD = ~0.165) in both groups. Power calculations were done with 3 different sample sizes in the two groups (MCMZ: 1500-900-500 versus DCMZ: 850-530-300). Note that the power to detect a difference in icc’s of the two groups is a function of the differences in values of the icc’s, and in the overall (or mean) value of the icc’s. That is, the effect size depends on the level of the icc and the difference in icc’s. We calculated the power given an alpha of 0.05/66 (Bonferroni correction for 66 phenotypes). The results relating to the continuous phenotypes are shown in Table S6 (Online Supplementary Materials I). For instance, given an icc of 0.6 in group1, the largest sample size (1500 versus 85) is adequate (power = 0.81) to detect a difference of 0.09 (i.e., icc = 0.69 in group 2), but the smaller sample sizes are not [power = 0.48 (for n = 900–530) and 0.19 for (n = 500–300)]. In contrast, given an icc of 0.8 in group 1, a difference of 0.08 (icc in group 2 of 0.88) is detectable with a power of 1 in the largest group and 0.93 in the smallest group. Thus for most traits we had adequate power to detect a differences of 0.08. In contrast, power calculations in the case of binary variables revealed that the power was too low to demonstrate the absence of any effect convincingly. For that reason the absence of any effect on binary variables should be interpreted with care.

## Discussion

By linking the data of the NTR with the data of PALGA, we obtained information on chorionicity based on pathology reports for more than 9000 twin pairs. This enabled us to examine the effect of chorion sharing on a large number of traits measured at different ages. As two-thirds of the MZ pairs share a chorion, the assumption of equal MZ and DZ (prenatal) environment has been seen as a possible threat to the validity of the classical twin design (Charney 2012; Phillips 1993). The general finding is that with respect to the wide range of phenotypes considered, the resemblance of MC MZ twins was no different from DC MZ twins. Power calculations revealed that for most traits we had enough power to demonstrate the absence of any effect given a correction of the alpha for multiple testing. Thus, intra-uterine environmental factors, as indexed by chorion sharing do not affect traits differently in MCMZ and DCMZ pairs. The few differences we did detect (which may be largely due to chance) were small and would produce only trivial bias in heritability estimates.

Intra-uterine environmental factors do influence the intra-pair similarity of MCMZ and DCMZ pairs for birth weight, weight during the first years of life, achieving motor milestone standing alone, externalizing behaviors at age 3, internalizing behaviors and anxiety at age 12, and autistic behavior at age 7. The difference in intra-class correlations (icc) between the two chorion groups ranged between 0.01 and 0.18. A difference in icc of around 0.03, as seen for example for externalizing behavior at age 3 years, has a relatively small effect on the heritability estimate. For instance, if we calculate the heritability based on the classical twin design as 2*(rMZ-rDZ), the heritability of externalizing behavior at age 3 will be 60 % [2*(0.84 (=average of DC and MC)−0.54)], and 56 % [2*(0.82–0.54)] in a design including only DCMZs. For internalizing behavior at age 12 years, with a difference of 0.11, the heritability will be 50 % [2*(0.71–0.46)] when including both types of twins and 38 % [2*(0.65–0.46)] when including only DC twins. For anxiety at age 12, the heritability will be 70 % [2*(0.71–0.36)] when both types were included and 52 % [2*(0.62–0.36)] when only DCMCs were included. Thus for some traits, ignoring the prenatal environment may slightly overestimate the influence of genetic effects, but the possibility of chance effects cannot be discounted.

Confirming earlier research, the weight measures in early life showed greater similarity in DCMZs than in MCMZs (Buzzard et al. 1983; Corey et al. 1979; Vlietinck et al. 1989). It is well known that MCMZ twins show more BWD than DCMZ twins (Gielen et al. 2008). MCMZ twins more often than DCMZ twins share their blood circulation, which may lead to unequal blood supply. This in turn results in an unequal supply of nutrients which gives rise to a difference in the birth weights of twins. As a consequence on average, birth weight of MCMZs is less similar than that of DCMZs. In agreement with this, we found that the percentage of twin pairs with a BWD larger than 20 % was larger in MC than in DC pairs.

In contrast to earlier reports, the mean birth weight of MCMZ twins was equal to that of DCMZ twins (Loos et al. 2001b, 2005). Reduced birth weight has been associated with peripheral cord insertion and fused placentas, and both conditions are more frequent in MCMZ than in DCMZ pairs (Loos et al. 2001b, 2005). One possible explanation for this contrast to the literature is that the placentas of the DCMZ twins in our study were more often abnormal placentas. We found that twins with successful record linkage had lower birth weight and higher rates of birth weight discordances than twins without successful linkage, and it is likely that the more abnormal placentas were more often submitted to the pathologist for evaluation. Another explanation is a possible selection bias in the NTR. Compared to a reference data set of all Dutch live-born twins, twins who are registered with the NTR have a somewhat higher birth weight and gestational age (Gielen et al. 2010).

The MCMZ—DCMZ differences of weight in the first years of life are probably due to the initial effect of chorion sharing on birth weight. The effect of chorion sharing on weight is in the same direction as birth weight: MCMZs, compared to DCMZs, display greater differences. However, after age 10, such differences are no longer present. Previous studies suggested little or no chorion effects on height and weight during childhood (Hur and Shin 2008). For adolescents, Gutknecht et al. (1999) reported larger intra-pair differences of weight in MCMZs than in DCMZs, but these findings were not confirmed by the larger Belgium study Loos et al. (2001b). Lower resemblance in MCMZs compared to DCMZs was also found for the achievement of motor milestone standing alone. As gestational age and birth weight are predictors for a delay of gross motor development (de Kieviet et al. 2009), the lower resemblance observed in MCMZs may be a consequence of the birth weight differences.

In contrast to the weight measures, the effects of chorion sharing on problem behaviors were in the reversed direction: greater resemblance of MCMZs than of DCMZs (with exception of the autism scale). Greater resemblance in MCMZs may be expected where blood supply is important. Sharing placenta and blood circulation may make the nutrimental and hormonal influences more similar in MCMZs. In turn, these nutrimental and hormonal influences may induce changes on tissue structure and function (Godfrey 2002; Loos et al. 2001b) with possible effect on physical health and psychopathology later in life (Harris and Seckl 2011). As the resemblance for a trait is greater in twins who share a chorion, the heritability may be overestimated. For 3 traits, the similarity in MCMZs was greater than the DCMZs (externalizing at age 3, internalizing and anxiety at age 12 years. Larger similarity for MCMZ pairs than for DCMZ pairs for behavioral dimensions like impulsivity, social incompetence, shyness, and internalizing/somatic symptoms have been reported in 5–6 years old twins (Sokol et al. 1995). However, later studies did not find significant chorion effects on temperaments ratings (Riese 1999), prosoical behaviors (Hur 2007), and on internalizing and externalizing behaviors (Wichers et al. 2002; Peerbooms et al. 2010). In our study the differences in DC and MC correlations ranged between 0.3 and 0.18, and previous studies may have been underpowered to detect small differences. Overall, the results do not suggest any appreciable overestimation of the heritability of problem behaviors. There were no significant effects of chorion sharing on cognition, as measured by standardized educational attainment and school performance tests. Larger within-pair similarity in 7-year-old MCMZs than in DCMZs were reported for IQ scores assessed by the WISC (Melnick et al. 1978), and for block design (WAIS) in adults (Rose et al. 1981) and in 10 years old twins (Spitz et al. 1996). Three years later, these authors could not replicate this finding in the same set of twin pairs, but reported larger within-pair differences for DCMZs for the Perceptive Organisation Index of the WISC-III (Gutknecht et al. 1999). In the largest study of IQ (Jacobs et al. 2001), a significant effect of chorionicity was reported for 2 out 12 subscales of the Wechsler Intelligence test. MCMZs were more similar than DCMZs, and chorion type explained 10–14 % of the variance. Differences in outcomes between our study and previous cognition studies may be explained by the use of different cognition measures, the number of twin pairs, the different classification methods of chorionicity, or by the use of different age groups.

The information obtained by record linkage may have limitations. Record linkage may be imperfect and results may include false-positive linkages. We examined the uniqueness of the provided identifiers, and it seems that a combination of birth date mother, birth date of offspring and mothers own last name (maiden name) is unique. Thus, the number of false-positives linkages is likely to be low in this study. The twins who were successfully linked may not be representative of all twins in the NTR or of the population of the Netherlands. About 44 % of the twins in the dataset were successfully linked. We found that placentas submitted to examination came from pairs with lower gestational age, lower birth weight, and more BWD compared to NTR twins without successful linkage. In addition, the coverage of twin and higher-order multiples placentas in the PALGA database decreased in the recent years. However, record linkage-procedures provided us with information on chorionicity on large numbers of twin pairs (9000 pairs in this study), who have been phenotyped for a large number of variables during childhood and adolescence. Earlier research showed that parental report for chorion status is not reliable (Derom et al. 2003), and by linkage with the national register, we obtained the chorion evaluation based on both gross and microscopic histopathological evaluation of placentas. Another issue is that we have only information on placental sharing in general, while twins may differ in other placental characteristics, such as unequal placental sharing, inter-twin placental proximity and competition of nutrients, the insertion and number of cord vessels, and chronic twin transfusion syndrome, which in turn may affect growth and developmental traits.

In testing the difference in MCMZ and DCMZ correlations, we recognize that misclassification of zygosity and of chorionicity may bias the correlations. To gauge the effect of such misclassification, we calculated the effect of zygosity and chorionicity misclassification on the MCMZ and DCMZ correlations. We estimated the chorionicity-misclassification probability, p(MC|DC), to equal 0.04. We fixed p(DC|MZ) to 0.05, as this probability is not identified given the present data. Furthermore, we assumed zygosity misclassification p(MZ|MZ) to be 0.97 (based on Rietveld et al. 2000) and p(DZ|DZ) in the DZ same sex twins to equal 0.937 (Rietveld et al. 2000). Finally we set the prior probabilities p(MZ) and p(DZ) to equal 1/3 and 2/3, which reflect these probabilities in the Dutch population (the NTR samples are representative in this respect). Given these results, we calculated the observed correlations in the MCMZ and DCMZ samples as a weighted combination of the DZ, DCMZ, and MCMZ correlations. We considered various values of these correlations, and found the difference in DCMZ versus MCMZ correlations was inflated. For instance, given the misclassification probabilities mentioned above, and true correlations of 0.2, 0.4, 0.4 (DZ, DCMZ, and MCMZ, respectively), the observed DCMZ and MCMZ correlations are 0.374 and 0.395, a difference of 0.021 (rather than 0). Similarly, given correlation true correlations of 0.2, 0.4, 0.45, the observed DCMZ and MCMZ correlations are 0.374 and 0.436, a difference of 0.062 (rather than 0.05). We refer to the supplemental documentation for further details and numerical results. We conclude that the two sources of misclassification result in an inflation of any difference in correlation. But as we observed relatively few significant differences we think that this inflation was not an appreciable source of false positives.

The information on chorionicity allows us to test the assumption of equal prenatal environment of MCMZ and DCMZ twins. We observed effects in both directions, with MCMZ twins less similar for birth weight and weight measures, and MCMZ twins more similar for problem behaviors. These differences can be attributed to the conditions of the (early) intra-uterine environment. Greater similarity of environments in MCMZs might occur as consequence of shared blood supply (e.g., virus infection) while less similarity could occur by competition for a limited food supply and by vascular anastomoses between the circulations of the fetuses. For females twins, an alternative explanation is possible: the pattern of X-inactivation is more similar in MCMZ than in DCMZs (Monteiro et al. 1998). Peerbooms et al. (2010) used chorion type as proxy for X-inactivation to examine the relation between X-inactivation and problem behavior and cognition. Given a causal relation, within-pair differences are expected to be larger in DCMZ than MCMZ female twin pairs, but not in male twin pairs. As this study did not reveal any significant results, there is limited evidence that the (dis)similarity in X-activation pattern play a role in behavior problems and cognition.

In conclusion, the findings of the present study indicate that the influence of the intra-uterine prenatal environment, as measured by chorion type, has a limited and small effect on MZ twin similarity with respect to measures of growth and behavioral development, and so a minor effect at best on the accuracy of heritability estimates obtain in the classical twin design (or any other design including MZ twins).

## Electronic supplementary material

Below is the link to the electronic supplementary material.

Supplementary material 1 (DOCX 87 kb)

Supplementary material 2 (DOCX 57 kb)
