# Supplementary material for: Chorionicity and Heritability Estimates from Twin Studies: The Prenatal Environment of Twins and Their Resemblance Across a Large Number of Traits
Source: Behav Genet. 2015 Sep 26;46:304–14. doi: 10.1007/s10519-015-9745-3 (PMC4858554; doi:10.1007/s10519-015-9745-3)
Supplement: Supplementary file 1 — Supplementary material 1 (DOCX 87 kb) [file 10519_2015_9745_MOESM1_ESM.docx]

Supplementary material I – Tables S1 – S6.

Chorionicity and heritability estimates from twin studies: The prenatal environment of twins and their resemblance across a large number of traits

Behavior Genetics

C.E.M. van Beijsterveldt^1^, L.I.H. Overbeek^2^, L. Rozendaal^4^, M.T.B. McMaster^1^, T.J. Glasner^1^, M. Bartels^1^, J.M. Vink^1^, N.G. Martin^3^, C.V. Dolan^1^, D.I. Boomsma^1^

^1^Department of Biological Psychology, VU University Amsterdam, Amsterdam, Netherlands; ^2^PALGA Foundation, Utrecht, Netherlands; ^3^Genetic Epidemiology Unit, Queensland Institute of Medical Research, Brisbane, Queensland, Australia; ^4^Department of Pathology, VU University Medical Centre, Amsterdam, Netherlands

Please address correspondence to: CEM van Beijsterveldt,

Department of Biological Psychology, VU University Amsterdam,

Van der Boechorststraat 1, 1081 BT Amsterdam, The Netherlands

E-mail: t.van.beijsterveldt@vu.nl

Fax: +31 20 5988832 / Tel: +31-20 5988787

Table S1: Overview of the number of twin births (twin pairs) in the Netherlands (CBS, 2013), estimated coverage by PALGA, and results of the records linkage between the NTR and PALGA data base (N no hit/N hit/% hit).

| Birth cohort | N twin pairs (CBS) | % coverage PALGA* | NTR  no hit  N | NTR  Hit  N | NTR  %hit |
| --- | --- | --- | --- | --- | --- |
| 1986 | 2194 |  | 74 | 39 | 35% |
| 1987 | 2250 |  | 309 | 182 | 37% |
| 1988 | 2346 |  | 275 | 213 | 44% |
| 1989 | 2480 |  | 333 | 269 | 45% |
| 1990 | 2690 |  | 344 | 290 | 46% |
| 1991 | 2794 | 58.95 | 343 | 328 | 49% |
| 1992 | 2789 | 53.93 | 390 | 323 | 45% |
| 1993 | 2825 | 59.68 | 389 | 403 | 51% |
| 1994 | 2818 | 58.87 | 440 | 406 | 48% |
| 1995 | 3070 | 51.11 | 479 | 448 | 48% |
| 1996 | 3197 | 52.36 | 559 | 493 | 47% |
| 1997 | 3245 | 52.27 | 600 | 514 | 46% |
| 1998 | 3556 | 47.10 | 673 | 528 | 44% |
| 1999 | 3634 | 50.25 | 703 | 528 | 43% |
| 2000 | 3639 | 46.72 | 627 | 523 | 45% |
| 2001 | 3695 | 46.98 | 648 | 475 | 42% |
| 2002 | 3707 | 47.61 | 657 | 534 | 45% |
| 2003 | 3616 | 48.04 | 655 | 553 | 46% |
| 2004 | 3523 | 44.37 | 706 | 507 | 42% |
| 2005 | 3448 | 42.02 | 547 | 406 | 42% |
| 2006 | 3210 | 42.27 | 430 | 299 | 41% |
| 2007 | 3023 | 40.03 | 465 | 315 | 40% |
| 2008 | 3189 | 38.51 | 397 | 251 | 39% |
| 2009 | 3153 | 34.70 | 468 | 259 | 36% |
| 2010 | 2974 | 35.07 | 567 | 294 | 34% |
| 2011 | 2825 | 33.13 | 265 | 148 | 36% |

Note. *estimate of the covering percentage of multiple placents by PALGA.

Table S2: Maternal and birth characteristics for the group with (hit) and without (no hit) successful record linkage.

|  |  |  | Mean | SD | t | p |
| --- | --- | --- | --- | --- | --- | --- |
| age mother | no hit | 11086 | 31.64 | 3.98 | 5.104 | <0.001 |
|  | hit | 9528 | 31.360 | 3.98 |  |  |
| gestational age | no hit | 10350 | 36.93 | 2.19 | 25.763 | <0.001 |
|  | hit | 8950 | 36.00 | 2.82 |  |  |
| birth weight 1^st^ born | no hit | 10238 | 2609.40 | 502.75 | 26.133 | <0.001 |
|  | hit | 8887 | 2404.54 | 581.32 |  |  |
| birth weight 2^nd^ 2^nd^born | no hit | 10217 | 2561.60 | 502.52 | 28.175 | <0.001 |
|  | hit | 8884 | 2338.29 | 592.81 |  |  |
|  | N no hit/  N hit |  |  |  |  |  |
|  |  |  | no hit | hit | chi^2^ |  |
| zygosity | 10905/9413 | % MZ | 24.5% | 41.1% | 671.800 | <0.001 |
| C-section | 8964/7775 | % C-section | 28.2% | 33.0% | 45.990 | <0.001 |
| educational level | 9254/7958 | Low | 26.1% | 27.6% | 4.983 | 0.083 |
|  |  | Mid | 43.3% | 42.2% |  |  |
|  |  | High | 30.6% | 30.2% |  |  |
| 20% BWD | 10252/8901 | % >20% BWD | 14.5% | 20.5% | 118.623 | <0.001 |
| conception | 10839/9362 | spontaneous | 71.8% | 76.8% | 77.352 | <0.001 |
|  |  | Hormones | 9.6% | 8.9% |  |  |
|  |  | IVF/ICSI | 18.6% | 14.3% |  |  |
| urbanization | 10894/9354 | very urban | 7.3% | 8.9% | 21.153 | <.001 |
|  |  | strong urban | 18.5% | 17.9% |  |  |
|  |  | moderate urban | 23.5% | 22.7% |  |  |
|  |  | low urban | 24.6% | 25.1% |  |  |
|  |  | not urban | 26.2% | 25.4% |  |  |

Note: 1st is first born; 2nd is second born; BWD is Birth Weight Discordance; MZ is monozygotic; C-section is caesarean section

Table S3: Overview of number and frequency of congenital and chronic diseases in 3-year old twins by type of twins. The p-values were the outcomes of chi-squared tests for frequency comparisons across types of twins.

|  | MCMA | MCDA | DCMZ | DZ | p |
| --- | --- | --- | --- | --- | --- |
| Asthma, chronic bronchitis or COPD | 14 (6.1%) | 253 (11.3%) | 168 (11.0%) | 338 (10.6%) | 0.343 |
| Infection of the nose or sinusitis | 2 (0.9%) | 47 (2.1%) | 24 (1.6%) | 71 (2.2%) | 0.508 |
| Serious skin disease or eczema | 20 (8.7%) | 171 (7.6%) | 136 (8.9%) | 231 (7.3%) | 0.402 |
| Serious intestinal disorders (longer than 3 months) | 3 (1.3%) | 22 (1.0%) | 16 (1.0%) | 34 (1.1%) | 0.977 |
| Chronic bladder infection | 0 (0.0%) | 4 (0.2%) | 3 (0.2%) | 6 (0.2%) | 0.992* |
| Epilepsy | 0 (0.0%) | 3 (0.1%) | 5 (0.3%) | 5 (0.2%) | 0.452* |
| Serious heart disease | 5 (2.2%) | 7 (0.3%) | 4 (0.3%) | 9 (0.3%) | 0.001 |
| Diabetes | 0 (0.0%) | 4 (0.2%) | 1 (0.1%) | 3 (0.1%) | 0.621 |
| Malignancy or cancer | 0 (0.0%) | 1 (0.1%) | 0 (0.0%) | 4 (0.1%) | - |
| Liver disease or cirrhosis | 0 (0.0%) | 0 (0.0%) | 0 (0.0%) | 1 (0.1%) | - |
| Serious kidney disease | 0 (0.0%) | 1 (0.1%) | 1 (0.0%) | 2 (0.1%) | 0.597* |
| Arthritis or chronic rheumatism (longer than 3 months) | 0 (0.0%) | 0 (0.0%) | 1 (0.1%) | 2 (0.1%) | - |
| Deafness or seriously hard of hearing | 1 (0.4%) | 7 (0.3%) | 1 (0.1%) | 7 (0.2%) | 0.478 |
| Blind or seriously visually impaired | 0 (0.0%) | 0 (0.0%) | 11 (0.7%) | 12 (0.4%) |  |
| Spasticity | 2 (0.9%) | 10 (0.4%) | 8 (0.5%) | 13 (0.4%) | 0.779 |
| Orthopedic disorder | 3 (1.3%) | 16 (0.7%) | 20 (1.3%) | 28 (0.9%) | 0.421 |
| Metabolic disorder | 0 (0.0%) | 5 (0.2%) | 5 (0.3%) | 5 (0.2%) | 0.571* |
| Other serious congenital or chronic diseases | 5 (2.5%) | 73 (3.6%) | 63 (4.6%) | 101 (3.5%) | 0.424 |

Note: MC refers to monochorionic; DC is dichorionic; MA is monoamniotic; DA is diamniotic; MZ is monozygotic; DZ is dizygotic. * refers to 2df test (MCMA not included)

Table S4: Weight, height, motor milestones, temperament, problem behaviors, cognition, personality and wellbeing in MCMZ and DCMZ pairs: number, mean, variance, covariance, correlations and test results for the comparison of MCMZs versus DCMZs by means of a likelihood-ratio test (1 df with the constraint that the variance for MCMZ and DCMZ are equal; 2df with no constraint for the variance of MCMZ and DCMZ twins), for alpha of .05/66 (~.00075). Bold indicates significant difference in intra-class correlations (icc’s) between the MCMZ and DCMZ groups.

|  | N 1^st^ borns | N 2^nd^ borns | VAR 1^st^ borns | VAR 2^nd^ borns | Covar | correlation | mean 1^st^ borns | mean 2^nd^ borns | icc |  | equal means | equal VAR | equal icc (1df) | equal icc (2 df) |
| --- | --- | --- | --- | --- | --- | --- | --- | --- | --- | --- | --- | --- | --- | --- |
| **Weight** |  |  |  |  |  |  |  |  |  |  |  |  |  |  |
| birth weight (in kg) | | | | | | | | | | | | | | |
| MCMZ | 2338 | 2342 | 0.30 | 0.32 | 0.23 | 0.75 | 2.38 | 2.33 | 0.74 | ∆Х^2^ | 0.35 | 0.27 | **31.27** | **31.54** |
| DCMZ | 1203 | 1204 | 0.32 | 0.32 | 0.26 | 0.81 | 2.38 | 2.35 | 0.81 | P | 0.55 | 0.61 | **0.00** | **0.00** |
| weight at 3 months (in kg) | | | | | | | | | | | | | | |
| MCMZ | 1532 | 1527 | 0.70 | 0.72 | 0.58 | 0.81 | 5.37 | 5.26 | 0.80 | ∆Х^2^ | 0.02 | 1.01 | **17.08** | **18.09** |
| DCMZ | 899 | 896 | 0.66 | 0.67 | 0.56 | 0.85 | 5.37 | 5.28 | 0.83 | P | 0.89 | 0.32 | **0.00** | **0.00** |
| weight at 6 months (in kg) | | | | | | | | | | | | | | |
| MCMZ | 1555 | 1556 | 0.86 | 0.87 | 0.70 | 0.81 | 7.12 | 6.99 | 0.79 | ∆Х^2^ | 1.46 | 0.60 | **15.85** | **16.45** |
| DCMZ | 883 | 883 | 0.82 | 0.81 | 0.68 | 0.84 | 7.05 | 6.96 | 0.83 | P | 0.23 | 0.44 | **0.00** | **0.00** |
| weight at 12 months (in kg) | | | | | | | | | | | | | | |
| MCMZ | 1236 | 1242 | 1.18 | 1.26 | 1.03 | 0.84 | 9.42 | 9.31 | 0.82 | ∆Х^2^ | 0.72 | 0.00 | 3.96 | 3.96 |
| DCMZ | 724 | 721 | 1.20 | 1.15 | 1.00 | 0.85 | 9.43 | 9.37 | 0.85 | P | 0.40 | 1.00 | 0.05 | 0.05 |
| weight at 18 months (in kg) | | | | | | | | | | | | | | |
| MCMZ | 859 | 858 | 1.66 | 1.75 | 1.43 | 0.84 | 11.06 | 10.91 | 0.83 | ∆Х^2^ | 0.63 | 1.61 | 0.28 | 1.89 |
| DCMZ | 460 | 461 | 1.55 | 1.64 | 1.33 | 0.83 | 11.10 | 10.98 | 0.82 | P | 0.43 | 0.20 | 0.60 | 0.60 |
| weight at 24 months (in kg) | | | | | | | | | | | | | | |
| MCMZ | 1097 | 1098 | 2.04 | 2.18 | 1.80 | 0.85 | 12.41 | 12.26 | 0.85 | ∆Х^2^ | 0.69 | 3.84 | 0.18 | 4.02 |
| DCMZ | 584 | 581 | 1.96 | 1.84 | 1.59 | 0.84 | 12.46 | 12.31 | 0.82 | P | 0.41 | 0.05 | 0.67 | 0.67 |
| weight at 30 months (in kg) | | | | | | | | | | | | | | |
| MCMZ | 448 | 449 | 2.51 | 2.63 | 2.16 | 0.84 | 13.48 | 13.37 | 0.84 | ∆Х^2^ | 0.37 | 0.43 | 3.93 | 4.36 |
| DCMZ | 276 | 280 | 2.50 | 2.39 | 2.13 | 0.87 | 13.45 | 13.25 | 0.86 | P | 0.54 | 0.51 | 0.05 | 0.05 |
| weight at 36 months (in kg) | | | | | | | | | | | | | | |
| MCMZ | 842 | 832 | 3.17 | 3.12 | 2.64 | 0.84 | 14.73 | 14.53 | 0.83 | ∆Х^2^ | 0.13 | 5.28 | 6.38 | 11.66 |
| DCMZ | 484 | 486 | 2.68 | 2.52 | 2.19 | 0.84 | 14.65 | 14.52 | 0.84 | P | 0.72 | 0.02 | 0.01 | 0.01 |
| weight at 48 months (in kg) | | | | | | | | | | | | | | |
| MCMZ | 766 | 760 | 4.42 | 4.47 | 3.78 | 0.85 | 16.49 | 16.31 | 0.84 | ∆Х^2^ | 0.00 | 0.72 | 8.27 | 8.99 |
| DCMZ | 486 | 480 | 4.18 | 4.18 | 3.66 | 0.88 | 16.49 | 16.31 | 0.87 | P | 0.96 | 0.40 | 0.004 | 0.011 |
| Weight at 60months (in kg) | | | | | | | | | | | | | | |
| MCMZ | 452 | 461 | 8.12 | 7.30 | 6.75 | 0.88 | 19.10 | 18.82 | 0.87 | ∆Х^2^ | 0.00 | 0.22 | **15.15** | **15.37** |
| DCMZ | 314 | 308 | 7.26 | 7.14 | 6.55 | 0.91 | 19.05 | 18.94 | 0.91 | P | 0.99 | 0.64 | **0.00** | **0.00** |
| Weight at 7 year (in kg) | | | | | | | | | | | | | | |
| MCMZ | 826 | 823 | 17.64 | 18.48 | 16.04 | 0.89 | 25.11 | 24.83 | 0.89 | ∆Х^2^ | 0.85 | **12.86** | **20.43** | **33.29** |
| DCMZ | 506 | 505 | 14.15 | 13.12 | 12.27 | 0.90 | 24.90 | 24.65 | 0.90 | P | 0.36 | **0.00** | **0.00** | **0.00** |
| weight at 10 year (in kg) | | | | | | | | | | | | | | |
| MCMZ | 710 | 709 | 36.66 | 35.79 | 32.42 | 0.90 | 33.82 | 33.34 | 0.89 | ∆Х^2^ | 0.96 | 0.00 | 3.13 | 3.13 |
| DCMZ | 446 | 444 | 36.48 | 35.21 | 32.52 | 0.91 | 33.39 | 33.03 | 0.91 | P | 0.33 | 0.96 | 0.08 | 0.08 |
| weight at 12 year (in kg) | | | | | | | | | | | | | | |
| MCMZ | 609 | 603 | 69.39 | 68.78 | 63.09 | 0.91 | 43.11 | 42.48 | 0.91 | ∆Х^2^ | 0.00 | 11.06 | 1.17 | 12.23 |
| DCMZ | 399 | 397 | 53.99 | 53.88 | 48.50 | 0.90 | 43.07 | 42.63 | 0.90 | P | 0.99 | 0.00 | 0.28 | 0.28 |
| **Height** | | | | | | | | | | | | | | |
| height at birth (in cm) | | | | | | | | | | | | | | |
| MCMZ | 1706 | 1701 | 14.76 | 15.04 | 12.85 | 0.86 | 45.88 | 45.65 | 0.86 | ∆Х^2^ | 0.58 | 0.03 | 5.37 | 5.40 |
| DCMZ | 856 | 860 | 15.04 | 14.97 | 13.20 | 0.88 | 45.90 | 45.86 | 0.88 | P | 0.45 | 0.87 | 0.02 | 0.07 |
| height at 3 months (in cm) | | | | | | | | | | | | | | |
| MCMZ | 1423 | 1410 | 9.35 | 10.06 | 8.03 | 0.83 | 58.20 | 57.97 | 0.82 | ∆Х^2^ | 0.03 | 0.01 | 0.00 | 0.01 |
| DCMZ | 837 | 831 | 9.87 | 9.68 | 8.07 | 0.83 | 58.18 | 58.06 | 0.82 | P | 0.87 | 0.92 | 0.99 | 0.99 |
| height at 6 months (in cm) | | | | | | | | | | | | | | |
| MCMZ | 1482 | 1472 | 8.47 | 9.48 | 7.71 | 0.86 | 65.25 | 65.01 | 0.85 | ∆Х^2^ | 0.21 | 0.02 | 0.06 | 0.08 |
| DCMZ | 851 | 847 | 8.92 | 8.95 | 7.63 | 0.85 | 65.13 | 65.02 | 0.84 | P | 0.65 | 0.89 | 0.80 | 0.80 |
| height at 12 months (in cm) | | | | | | | | | | | | | | |
| MCMZ | 1200 | 1207 | 8.72 | 9.70 | 8.07 | 0.88 | 74.65 | 74.51 | 0.87 | ∆Х^2^ | 0.12 | 0.50 | 3.91 | 4.41 |
| DCMZ | 704 | 704 | 8.52 | 8.20 | 7.06 | 0.84 | 74.63 | 74.60 | 0.84 | P | 0.73 | 0.48 | 0.05 | 0.05 |
| height at 18 months (in cm) | | | | | | | | | | | | | | |
| MCMZ | 854 | 845 | 11.04 | 11.39 | 9.95 | 0.89 | 81.63 | 81.39 | 0.89 | ∆Х^2^ | 0.08 | 2.93 | 0.84 | 3.78 |
| DCMZ | 455 | 456 | 9.91 | 10.57 | 8.87 | 0.87 | 81.56 | 81.53 | 0.86 | P | 0.78 | 0.09 | 0.36 | 0.36 |
| height at 24 months (in cm) | | | | | | | | | | | | | | |
| MCMZ | 1086 | 1083 | 12.07 | 12.96 | 11.34 | 0.91 | 87.50 | 87.33 | 0.91 | ∆Х^2^ | 1.70 | 4.13 | 7.53 | 11.67 |
| DCMZ | 573 | 576 | 11.40 | 11.06 | 9.80 | 0.87 | 87.70 | 87.60 | 0.87 | P | 0.19 | 0.04 | 0.01 | 0.01 |
| height at 30 months (in cm) | | | | | | | | | | | | | | |
| MCMZ | 440 | 443 | 14.76 | 14.41 | 13.24 | 0.91 | 92.21 | 92.18 | 0.91 | ∆Х^2^ | 0.03 | 0.00 | 0.71 | 0.72 |
| DCMZ | 272 | 278 | 14.59 | 14.14 | 13.15 | 0.92 | 92.37 | 92.16 | 0.91 | P | 0.85 | 0.97 | 0.40 | 0.40 |
| height at 36 months (in cm) | | | | | | | | | | | | | | |
| MCMZ | 836 | 825 | 15.64 | 16.19 | 14.27 | 0.90 | 96.91 | 96.82 | 0.90 | ∆Х^2^ | 0.01 | 0.25 | 0.04 | 0.29 |
| DCMZ | 484 | 483 | 15.50 | 15.07 | 13.62 | 0.89 | 96.99 | 96.80 | 0.89 | P | 0.92 | 0.62 | 0.85 | 0.85 |
| height at 48 months (in cm) | | | | | | | | | | | | | | |
| MCMZ | 779 | 772 | 19.74 | 21.24 | 18.83 | 0.92 | 103.73 | 103.63 | 0.92 | ∆Х^2^ | 0.21 | 1.15 | 2.65 | 3.80 |
| DCMZ | 494 | 481 | 18.84 | 18.90 | 16.97 | 0.90 | 103.61 | 103.52 | 0.90 | P | 0.65 | 0.28 | 0.10 | 0.10 |
| height at 60 months (in cm) | | | | | | | | | | | | | | |
| MCMZ | 463 | 470 | 32.82 | 33.64 | 31.23 | 0.94 | 112.52 | 112.30 | 0.94 | ∆Х^2^ | 0.01 | 0.30 | 0.42 | 0.72 |
| DCMZ | 314 | 312 | 31.47 | 28.93 | 28.32 | 0.94 | 112.52 | 112.38 | 0.94 | P | 0.91 | 0.59 | 0.52 | 0.52 |
| height at 7 year (in cm) | | | | | | | | | | | | | | |
| MCMZ | 818 | 817 | 38.02 | 38.73 | 36.03 | 0.94 | 127.52 | 127.35 | 0.94 | ∆Х^2^ | 0.29 | 3.68 | 0.51 | 4.19 |
| DCMZ | 515 | 515 | 33.45 | 32.61 | 30.85 | 0.93 | 127.39 | 127.12 | 0.93 | P | 0.59 | 0.06 | 0.48 | 0.48 |
| height at 10 year (in cm) | | | | | | | | | | | | | | |
| MCMZ | 721 | 721 | 47.52 | 49.27 | 45.50 | 0.94 | 142.73 | 142.50 | 0.94 | ∆Х^2^ | 0.09 | 0.31 | 1.59 | 1.90 |
| DCMZ | 446 | 445 | 51.31 | 50.29 | 47.56 | 0.94 | 142.60 | 142.39 | 0.94 | P | 0.77 | 0.58 | 0.21 | 0.21 |
| height at 12 year (in cm) | | | | | | | | | | | | | | |
| MCMZ | 613 | 611 | 61.98 | 65.57 | 59.29 | 0.93 | 156.11 | 156.02 | 0.93 | ∆Х^2^ | 0.35 | 2.10 | 2.12 | 4.22 |
| DCMZ | 394 | 393 | 55.22 | 56.05 | 51.77 | 0.93 | 156.53 | 156.19 | 0.93 | P | 0.55 | 0.15 | 0.15 | 0.15 |
| **motor miles attainment (age 2)** | | | | | | | | | | | | | | |
| Turning | | | | | | | | | | | | | | |
| MCMZ | 1729 | 1727 | 2.64 | 2.54 | 2.25 | 0.87 | 6.33 | 6.34 | 0.87 | ∆Х^2^ | 0.46 | 0.07 | 1.97 | 2.03 |
| DCMZ | 997 | 999 | 2.54 | 2.64 | 2.27 | 0.88 | 6.26 | 6.33 | 0.88 | P | 0.50 | 0.80 | 0.16 | 0.16 |
| Sitting | | | | | | | | | | | | | | |
| MCMZ | 1764 | 1765 | 3.45 | 3.61 | 3.28 | 0.93 | 8.96 | 9.00 | 0.93 | ∆Х^2^ | 2.50 | 0.86 | 6.56 | 7.43 |
| DCMZ | 1011 | 1012 | 3.19 | 3.40 | 3.02 | 0.92 | 8.84 | 8.91 | 0.92 | P | 0.11 | 0.35 | 0.01 | 0.01 |
| Crawling | | | | | | | | | | | | | | |
| MCMZ | 1721 | 1728 | 4.90 | 4.80 | 4.35 | 0.90 | 10.34 | 10.32 | 0.90 | ∆Х^2^ | 3.97 | 0.33 | 4.31 | 4.64 |
| DCMZ | 993 | 986 | 4.61 | 4.73 | 4.11 | 0.88 | 10.15 | 10.18 | 0.88 | P | 0.05 | 0.57 | 0.04 | 0.04 |
| Standing | | | | | | | | | | | | | | |
| MCMZ | 1754 | 1753 | 6.94 | 6.89 | 6.29 | 0.91 | 12.83 | 12.86 | 0.91 | ∆Х^2^ | 1.51 | 3.71 | **24.86** | **28.57** |
| DCMZ | 1010 | 1012 | 7.56 | 7.72 | 7.17 | 0.94 | 12.73 | 12.73 | 0.94 | P | 0.22 | 0.05 | **0.00** | **0.00** |
| Walking | | | | | | | | | | | | | | |
| MCMZ | 1812 | 1809 | 5.63 | 5.59 | 5.14 | 0.92 | 15.35 | 15.39 | 0.92 | ∆Х^2^ | 1.14 | 1.51 | 10.94 | 12.45 |
| DCMZ | 1037 | 1037 | 6.03 | 5.99 | 5.44 | 0.91 | 15.23 | 15.32 | 0.91 | P | 0.29 | 0.22 | 0.001 | 0.001 |
| **temperament (age 2)** | | | | | | | | | | | | | | |
| Shyness | | | | | | | | | | | | | | |
| MCMZ | 269 | 270 | 12.04 | 12.34 | 7.22 | 0.59 | 11.72 | 11.77 | 0.59 | ∆Х^2^ | 0.11 | 0.10 | 0.03 | 0.13 |
| DCMZ | 66 | 66 | 11.99 | 11.01 | 6.48 | 0.56 | 11.70 | 12.05 | 0.56 | P | 0.74 | 0.76 | 0.86 | 0.86 |
| Active | | | | | | | | | | | | | | |
| MCMZ | 270 | 267 | 11.76 | 9.46 | 5.32 | 0.51 | 18.34 | 18.84 | 0.48 | ∆Х^2^ | 0.16 | 0.42 | 2.47 | 2.89 |
| DCMZ | 66 | 66 | 8.89 | 10.04 | 2.58 | 0.27 | 18.76 | 18.68 | 0.28 | P | 0.69 | 0.52 | 0.12 | 0.12 |
| Emotionality | | | | | | | | | | | | | | |
| MCMZ | 269 | 270 | 18.19 | 18.25 | 13.73 | 0.75 | 13.00 | 13.25 | 0.75 | ∆Х^2^ | 0.11 | 0.71 | 2.73 | 3.44 |
| DCMZ | 67 | 66 | 16.12 | 15.47 | 9.75 | 0.62 | 13.22 | 13.38 | 0.62 | P | 0.74 | 0.40 | 0.10 | 0.10 |
| Sociability | | | | | | | | | | | | | | |
| MCMZ | 269 | 269 | 9.29 | 8.56 | 4.51 | 0.51 | 17.25 | 17.47 | 0.50 | ∆Х^2^ | 0.89 | 1.80 | 0.02 | 1.82 |
| DCMZ | 66 | 66 | 7.49 | 6.95 | 3.22 | 0.45 | 17.74 | 17.59 | 0.44 | P | 0.35 | 0.18 | 0.89 | 0.89 |
| **Problem behavior (age3 –age12)** | | | | | | | | | | | | | | |
| internalizing age 3 | | | | | | | | | | | | | | |
| MCMZ | 1545 | 1538 | 15.85 | 15.41 | 11.79 | 0.75 | 4.75 | 4.60 | 0.76 | ∆Х^2^ | 0.03 | 0.01 | 10.14 | 10.15 |
| DCMZ | 931 | 925 | 15.43 | 16.53 | 11.33 | 0.71 | 4.78 | 4.64 | 0.71 | P | 0.86 | 0.90 | 0.001 | 0.002 |
| internalizing age 7 | | | | | | | | | | | | | | |
| MCMZ | 1100 | 1090 | 21.94 | 17.99 | 13.20 | 0.66 | 4.77 | 4.39 | 0.66 | ∆Х^2^ | 3.25 | 0.23 | 0.19 | 0.42 |
| DCMZ | 666 | 660 | 20.45 | 17.95 | 12.27 | 0.64 | 4.36 | 4.04 | 0.64 | P | 0.07 | 0.63 | 0.66 | 0.66 |
| internalizing age 10 | | | | | | | | | | | | | | |
| MCMZ | 896 | 887 | 28.85 | 24.19 | 18.33 | 0.69 | 5.07 | 4.69 | 0.69 | ∆Х^2^ | 0.67 | 0.18 | 0.29 | 0.47 |
| DCMZ | 554 | 547 | 26.83 | 23.06 | 17.12 | 0.69 | 4.76 | 4.50 | 0.69 | P | 0.41 | 0.67 | 0.59 | 0.59 |
| internalizing age 12 | | | | | | | | | | | | | | |
| MCMZ | 780 | 775 | 29.99 | 27.34 | 21.70 | 0.76 | 4.51 | 4.24 | 0.76 | ∆Х^2^ | 0.37 | 9.78 | 5.68 | 15.46 |
| DCMZ | 472 | 465 | 24.35 | 21.60 | 14.81 | 0.65 | 4.22 | 4.16 | 0.65 | P | 0.55 | 0.00 | 0.02 | 0.00 |
| externalizing age 3 | | | | | | | | | | | | | | |
| MCMZ | 1542 | 1530 | 92.62 | 94.59 | 79.94 | 0.85 | 16.15 | 15.58 | 0.85 | ∆Х^2^ | 0.49 | 1.12 | **20.05** | **21.16** |
| DCMZ | 933 | 924 | 93.37 | 103.63 | 80.59 | 0.82 | 16.42 | 15.90 | 0.82 | P | 0.49 | 0.29 | **0.00** | **0.00** |
| externalizing age 7 | | | | | | | | | | | | | | |
| MCMZ | 1107 | 1100 | 44.68 | 44.16 | 36.37 | 0.82 | 7.72 | 7.37 | 0.81 | ∆Х^2^ | 2.38 | 0.26 | 0.63 | 0.89 |
| DCMZ | 673 | 670 | 44.93 | 42.75 | 35.36 | 0.81 | 7.28 | 6.94 | 0.80 | P | 0.12 | 0.61 | 0.43 | 0.43 |
| externalizing age 10 | | | | | | | | | | | | | | |
| MCMZ | 904 | 899 | 45.65 | 44.19 | 36.97 | 0.82 | 7.09 | 6.62 | 0.82 | ∆Х^2^ | 3.25 | 1.91 | 2.43 | 4.35 |
| DCMZ | 558 | 555 | 39.66 | 41.15 | 33.39 | 0.83 | 6.44 | 6.09 | 0.82 | P | 0.07 | 0.17 | 0.12 | 0.12 |
| externalizing age 12 | | | | | | | | | | | | | | |
| MCMZ | 783 | 780 | 38.17 | 32.08 | 28.35 | 0.81 | 5.89 | 5.35 | 0.80 | ∆Х^2^ | 2.31 | 1.97 | 5.47 | 7.44 |
| DCMZ | 476 | 470 | 33.04 | 27.34 | 24.67 | 0.82 | 5.31 | 5.01 | 0.82 | P | 0.13 | 0.16 | 0.02 | 0.02 |
| anxiety age 3 | | | | | | | | | | | | | | |
| MCMZ | 1546 | 1541 | 9.86 | 9.55 | 7.05 | 0.73 | 3.61 | 3.47 | 0.73 | ∆Х^2^ | 0.02 | 0.64 | 3.56 | 4.19 |
| DCMZ | 932 | 925 | 9.03 | 9.93 | 6.54 | 0.69 | 3.62 | 3.51 | 0.69 | P | 0.89 | 0.43 | 0.06 | 0.06 |
| anxiety age 7 | | | | | | | | | | | | | | |
| MCMZ | 1112 | 1108 | 7.04 | 6.24 | 3.62 | 0.55 | 2.21 | 2.05 | 0.54 | ∆Х^2^ | 2.05 | 0.85 | 0.14 | 0.99 |
| DCMZ | 673 | 670 | 6.99 | 6.93 | 3.81 | 0.55 | 2.03 | 1.90 | 0.55 | P | 0.15 | 0.36 | 0.71 | 0.71 |
| anxiety age 10 | | | | | | | | | | | | | | |
| MCMZ | 909 | 905 | 10.91 | 9.56 | 6.31 | 0.62 | 2.62 | 2.42 | 0.61 | ∆Х^2^ | 2.34 | 3.33 | 1.40 | 4.74 |
| DCMZ | 561 | 557 | 9.36 | 8.72 | 5.55 | 0.61 | 2.33 | 2.24 | 0.61 | P | 0.13 | 0.07 | 0.24 | 0.24 |
| anxiety age 12 | | | | | | | | | | | | | | |
| MCMZ | 783 | 780 | 10.65 | 10.04 | 7.34 | 0.71 | 2.34 | 2.27 | 0.71 | ∆Х^2^ | 0.95 | 4.85 | **21.19** | **26.03** |
| DCMZ | 477 | 471 | 9.04 | 9.03 | 4.83 | 0.54 | 2.15 | 2.16 | 0.53 | P | 0.33 | 0.03 | **0.000** | **0.00** |
| autism age 7 | | | | | | | | | | | | | | |
| MCMZ | 1111 | 1109 | 3.69 | 3.44 | 1.99 | 0.56 | 1.51 | 1.47 | 0.55 | ∆Х^2^ | 1.52 | 0.01 | **13.99** | **14.00** |
| DCMZ | 673 | 670 | 3.93 | 3.24 | 2.38 | 0.67 | 1.47 | 1.32 | 0.65 | P | 0.22 | 0.91 | **0.00** | **0.001** |
| autism age 10 | | | | | | | | | | | | | | |
| MCMZ | 909 | 908 | 3.41 | 4.00 | 2.14 | 0.58 | 1.49 | 1.53 | 0.57 | ∆Х^2^ | 5.04 | 5.34 | 2.76 | 8.09 |
| DCMZ | 560 | 558 | 3.06 | 3.33 | 1.87 | 0.59 | 1.29 | 1.33 | 0.58 | P | 0.03 | 0.02 | 0.10 | 0.10 |
| autism age 12 | | | | | | | | | | | | | | |
| MCMZ | 783 | 781 | 3.45 | 3.21 | 2.02 | 0.61 | 1.33 | 1.32 | 0.60 | ∆Х^2^ | 0.36 | 0.15 | 0.36 | 0.51 |
| DCMZ | 476 | 473 | 3.28 | 3.17 | 1.86 | 0.58 | 1.29 | 1.25 | 0.58 | P | 0.55 | 0.70 | 0.55 | 0.55 |
| ADHD-index age 7 | | | | | | | | | | | | | | |
| MCMZ | 675 | 670 | 49.79 | 48.87 | 36.41 | 0.74 | 7.38 | 7.03 | 0.73 | ∆Х^2^ | 0.57 | 3.79 | 0.70 | 4.48 |
| DCMZ | 430 | 429 | 42.06 | 43.89 | 31.26 | 0.73 | 7.15 | 6.86 | 0.71 | P | 0.45 | 0.05 | 0.40 | 0.40 |
| ADHD-index age 10 | | | | | | | | | | | | | | |
| MCMZ | 690 | 690 | 43.96 | 49.36 | 33.26 | 0.71 | 7.45 | 7.37 | 0.71 | ∆Х^2^ | 3.59 | 1.14 | 3.56 | 4.71 |
| DCMZ | 424 | 423 | 39.18 | 48.67 | 32.76 | 0.75 | 6.50 | 6.88 | 0.73 | P | 0.06 | 0.29 | 0.06 | 0.06 |
| ADHD-index age 12 | | | | | | | | | | | | | | |
| MCMZ | 685 | 683 | 39.20 | 40.85 | 31.72 | 0.79 | 6.08 | 6.08 | 0.78 | ∆Х^2^ | 1.02 | 0.02 | 0.87 | 0.89 |
| DCMZ | 424 | 419 | 39.35 | 41.61 | 32.82 | 0.81 | 5.72 | 5.81 | 0.80 | P | 0.31 | 0.90 | 0.35 | 0.35 |
| **cognition childhood** | | | | | | | | | | | | | | |
| CITO final score | | | | | | | | | | | | | | |
| MCMZ | 627 | 631 | 68.00 | 72.06 | 55.33 | 0.79 | 538.19 | 537.64 | 0.80 | ∆Х^2^ | 0.24 | 1.05 | 2.70 | 3.75 |
| DCMZ | 382 | 371 | 68.01 | 71.57 | 57.21 | 0.82 | 538.16 | 538.04 | 0.82 | P | 0.62 | 0.31 | 0.10 | 0.10 |
| Math | | | | | | | | | | | | | | |
| MCMZ | 527 | 524 | 1.17 | 1.30 | 0.91 | 0.74 | 3.86 | 3.80 | 0.74 | ∆Х^2^ | 0.66 | 0.18 | 0.08 | 0.26 |
| DCMZ | 329 | 328 | 1.31 | 1.24 | 0.94 | 0.74 | 3.75 | 3.81 | 0.74 | P | 0.42 | 0.67 | 0.78 | 0.78 |
| Language | | | | | | | | | | | | | | |
| MCMZ | 492 | 485 | 1.04 | 1.01 | 0.75 | 0.73 | 3.76 | 3.78 | 0.72 | ∆Х^2^ | 0.98 | 1.03 | 0.77 | 1.80 |
| DCMZ | 298 | 308 | 1.08 | 0.86 | 0.73 | 0.75 | 3.82 | 3.87 | 0.73 | P | 0.32 | 0.31 | 0.38 | 0.38 |
| Reading | | | | | | | | | | | | | | |
| MCMZ | 438 | 446 | 1.67 | 1.51 | 1.22 | 0.77 | 3.59 | 3.69 | 0.76 | ∆Х^2^ | 2.46 | 0.11 | 0.64 | 0.75 |
| DCMZ | 271 | 270 | 1.44 | 1.60 | 1.12 | 0.74 | 3.74 | 3.81 | 0.73 | P | 0.12 | 0.74 | 0.43 | 0.43 |
| Sports | | | | | | | | | | | | | | |
| MCMZ | 336 | 346 | 0.79 | 0.79 | 0.56 | 0.71 | 4.02 | 4.04 | 0.69 | ∆Х^2^ | 0.33 | 0.02 | 5.64 | 5.67 |
| DCMZ | 203 | 215 | 0.65 | 0.71 | 0.52 | 0.76 | 4.02 | 3.96 | 0.78 | P | 0.57 | 0.88 | 0.02 | 0.02 |
| **Adolescence** | | | | | | | | | | | | | | |
| **Problem behavior (age14- age18)** | | | | | | | | | | | | | | |
| internalizing YSR | | | | | | | | | | | | | | |
| MCMZ | 623 | 635 | 54.62 | 52.91 | 27.68 | 0.52 | 8.63 | 9.09 | 0.48 | ∆Х^2^ | 0.05 | 0.10 | 3.05 | 3.15 |
| DCMZ | 385 | 375 | 57.73 | 51.38 | 31.94 | 0.59 | 9.20 | 8.78 | 0.57 | P | 0.83 | 0.75 | 0.08 | 0.08 |
| externalizing YSR | | | | | | | | | | | | | | |
| MCMZ | 640 | 650 | 25.93 | 29.02 | 14.94 | 0.55 | 7.44 | 7.91 | 0.54 | ∆Х^2^ | 0.19 | 0.22 | 0.58 | 0.81 |
| DCMZ | 398 | 386 | 25.21 | 25.47 | 13.92 | 0.55 | 7.99 | 7.67 | 0.56 | P | 0.66 | 0.64 | 0.45 | 0.45 |
| **personality** | | | | | | | | | | | | | | |
| NEO-neuroticism | | | | | | | | | | | | | | |
| MCMZ | 354 | 345 | 49.05 | 63.22 | 24.22 | 0.44 | 31.09 | 31.09 | 0.40 | ∆Х^2^ | 0.39 | 0.01 | 1.80 | 1.81 |
| DCMZ | 219 | 221 | 59.47 | 55.17 | 30.25 | 0.53 | 31.34 | 31.32 | 0.49 | P | 0.53 | 0.93 | 0.18 | 0.18 |
| NEO-extraversion | | | | | | | | | | | | | | |
| MCMZ | 354 | 345 | 34.74 | 43.26 | 18.86 | 0.49 | 42.62 | 42.66 | 0.48 | ∆Х^2^ | 0.94 | 4.49 | 1.72 | 6.21 |
| DCMZ | 219 | 221 | 30.23 | 32.54 | 15.80 | 0.50 | 43.12 | 43.07 | 0.50 | P | 0.33 | 0.03 | 0.19 | 0.19 |
| NEO-openness | | | | | | | | | | | | | | |
| MCMZ | 354 | 345 | 33.33 | 29.95 | 12.80 | 0.41 | 35.43 | 35.35 | 0.40 | ∆Х^2^ | 0.16 | 0.31 | 6.76 | 7.07 |
| DCMZ | 219 | 221 | 31.23 | 31.53 | 17.94 | 0.57 | 35.49 | 35.65 | 0.56 | P | 0.69 | 0.58 | 0.009 | 0.029 |
| NEO-agreeableness | | | | | | | | | | | | | | |
| MCMZ | 354 | 345 | 20.07 | 24.66 | 9.78 | 0.44 | 43.97 | 43.82 | 0.39 | ∆Х^2^ | 0.76 | 0.00 | 0.53 | 0.53 |
| DCMZ | 219 | 221 | 19.85 | 25.13 | 11.09 | 0.50 | 43.39 | 43.81 | 0.45 | P | 0.38 | 0.98 | 0.47 | 0.47 |
| NEO-conscientiousness | | | | | | | | | | | | | | |
| MCMZ | 354 | 344 | 31.85 | 32.17 | 13.40 | 0.42 | 43.44 | 43.76 | 0.42 | ∆Х^2^ | 1.12 | 0.46 | 0.02 | 0.48 |
| DCMZ | 219 | 221 | 35.77 | 28.91 | 14.16 | 0.44 | 42.85 | 43.38 | 0.41 | P | 0.29 | 0.50 | 0.90 | 0.90 |
| **wellbeing** | | | | | | | | | | | | | | |
| subjective wellbeing | | | | | | | | | | | | | | |
| MCMZ | 643 | 652 | 0.84 | 0.95 | 0.40 | 0.45 | 0.02 | -0.02 | 0.45 | ∆Х^2^ | 0.92 | 3.35 | 0.03 | 3.38 |
| DCMZ | 396 | 387 | 0.72 | 0.78 | 0.30 | 0.40 | 0.06 | 0.04 | 0.41 | P | 0.34 | 0.07 | 0.86 | 0.86 |

^Note: MCMZ refers to monochorionic monozygotic twin pairs. DCMZ refers to monozygotic twin pairs.^

Table S5: Asthma and related diseases, and hand preference in MCMZ and DCMZ pairs: Number, thresholds, intraclass correlations (icc’s), and test results for the comparison of MCMZs vs DCMZs by means of a likelihood ratio test (1 df with the constraint that the variance for MCMZ and DCMZ are equal; 2df with no constraint for the variance of MCMZ and DCMZ twins), given an alpha of .05/5 (~.01).

|  |  | N 1st borns | N 2nd borns | Threshold 1st borns | Threshold 2nd borns | icc |  | test of equal thresholds | test of equal icc |
| --- | --- | --- | --- | --- | --- | --- | --- | --- | --- |
| bronchitis | MCMZ | 1260 | 1249 | 0.91 | 0.858 | 0.946 | ∆Х^2^ | 0.057 | 3.585 |
|  | DCMZ | 784 | 778 | 0.893 | 0.842 | 0.907 | p | 0.811 | 0.167 |
| pneumonia | MCMZ | 1230 | 1223 | 1.27 | 1.33 | 0.851 | ∆Х^2^ | 0.393 | 0.213 |
|  | DCMZ | 776 | 773 | 1.34 | 1.33 | 0.866 | p | 0.531 | 0.899 |
| asthma | MCMZ | 1244 | 1234 | 1.31 | 1.36 | 0.948 | ∆Х^2^ | 0.209 | 2.809 |
|  | DCMZ | 778 | 778 | 1.3 | 1.3 | 0.904 | p | 0.647 | 0.246 |
| eczema | MCMZ | 1240 | 1235 | 0.842 | 0.836 | 0.857 | ∆Х^2^ | 1.693 | 0.056 |
|  | DCMZ | 781 | 782 | 0.929 | 0.915 | 0.843 | p | 0.193 | 0.972 |
| hand preference | MCMZ | 1719 | 1717 | 0.755 | 0.751 | 0.455 | ∆Х^2^ | 0.121 | 0.309 |
|  | DCMZ | 1066 | 1061 | 0.735 | 0.802 | 0.488 | p | 0.728 | 0.857 |

Note: MC refers to monochorionic monozygotic twin pairs; DC refers to monozygotic twin pairs; icc is intraclass correlation

Table S6: Power (probability) for detecting the difference between intraclass correlation in the MCMZ group (icc1) and DCMZ group (icc2) for 3 different sample sizes by means of a likelihood ratio test (1 df), given an alpha of .05/66 (~.00075)

| icc1 MCMZ | icc2  DCMZ | N1=1500 and N2=850 | N1=900 and N2=530 | N1=500 and N2=300 |
| --- | --- | --- | --- | --- |
| 0.6 | 0.69 | 0.8089 | 0.4842 | 0.1917 |
| 0.6 | 0.70 | 0.9203 | 0.6478 | 0.2886 |
| 0.7 | 0.77 | 0.8415 | 0.5240 | 0.2128 |
| 0.7 | 0.78 | 0.9570 | 0.7331 | 0.3538 |
| 0.7 | 0.79 | 0.9931 | 0.8863 | 0.5254 |
| 0.7 | 0.80 | 0.9994 | 0.9653 | 0.6988 |
| 0.8 | 0.85 | 0.9071 | 0.6230 | 0.2720 |
| 0.8 | 0.86 | 0.9923 | 0.8806 | 0.5167 |
| 0.8 | 0.87 | 0.9998 | 0.9820 | 0.7680 |
| 0.8 | 0.88 | 1.0000 | 0.9990 | 0.9293 |
| 0.8 | 0.89 | 1.0000 | 1.0000 | 0.9882 |
| 0.8 | 0.90 | 1.0000 | 1.0000 | 0.9991 |
| 0.9 | 0.93 | 0.9919 | 0.8775 | 0.5121 |
| 0.9 | 0.94 | 1.0000 | 0.9989 | 0.9274 |
| 0.9 | 0.95 | 1.0000 | 1.0000 | 0.9990 |
| 0.9 | 0.96 | 1.0000 | 1.0000 | 1.0000 |
| 0.9 | 0.97 | 1.0000 | 1.0000 | 1.0000 |
| 0.9 | 0.98 | 1.0000 | 1.0000 | 1.0000 |
| 0.9 | 0.99 | 1.0000 | 1.0000 | 1.0000 |
