# Supplementary material for: Chorionicity and Heritability Estimates from Twin Studies: The Prenatal Environment of Twins and Their Resemblance Across a Large Number of Traits
Source: Behav Genet. 2015 Sep 26;46:304–14. doi: 10.1007/s10519-015-9745-3 (PMC4858554; doi:10.1007/s10519-015-9745-3)
Supplement: Supplementary file 2 — Supplementary material 2 (DOCX 57 kb) [file 10519_2015_9745_MOESM2_ESM.docx]

Supplementary material II – Sensitivity analysis

Chorionicity and heritability estimates from twin studies: The prenatal environment of twins and their resemblance across a large number of traits

Behavior Genetics

C.E.M. van Beijsterveldt^1^, L.I.H. Overbeek^2^, L. Rozendaal^4^, M.T.B. McMaster^1^, T.J. Glasner^1^, M. Bartels^1^, J.M. Vink^1^, N.G. Martin^3^, C.V. Dolan^1^, D.I. Boomsma^1^

^1^Department of Biological Psychology, VU University Amsterdam, Amsterdam, Netherlands; ^2^PALGA Foundation, Utrecht, Netherlands; ^3^Genetic Epidemiology Unit, Queensland Institute of Medical Research, Brisbane, Queensland, Australia; ^4^Department of Pathology, VU University Medical Centre, Amsterdam, Netherlands

Please address correspondence to: CEM van Beijsterveldt,

Department of Biological Psychology, VU University Amsterdam,

Van der Boechorststraat 1, 1081 BT Amsterdam, The Netherlands

E-mail: t.van.beijsterveldt@vu.nl

Fax: +31 20 5988832 / Tel: +31-20 5988787

Sensitivity analysis: effects on phenotypic MCMZ and MZDC correlations of zygosity and chorionicity misclassification.

*Summary: we gauge the effect of zygosity and chorionicity misclassification on the MCMZ and DCMZ phenotypic correlations. Misclassification probabilities are prob(DZ|MZ)=.03, prob(MZ|DZ)=.063, prob(MC|DC)=.04, and prob(DC|MC)=.05. Misclassification gives rise to an upwards bias in the difference in the MCMZ and DCMZ correlations. The fact that we observed relatively few significant differences in these correlations suggests that the upwards bias did not result in many false positives (i.e., inferring a differences in correlation, where there is none).*

The aim of the supplemental analyses detailed here was to assess the effect of zygosity and chorionicity misclassification on the phenotypic correlation of monochorionic (MC) and dichorionic (DC) monozygotic (MZ) twins. The data used in these analyses are shown in Table S-1, in which the numbers of DC and MC twin pairs are given by zygosity and sex. The number of MC twin pairs is broken down by amnionicity (i.e., monoamniotic (MA) and diamniotic (DA)).

*Table S-1: Classification of twin pairs according to chorionicity and amnionicity. The number within the brackets represents the misclassified cases. Note that the MC counts are broken down in the adjacent columns (MCMA, MCDA and Missing).*

|  | Chorion | | Amnion | | |
| --- | --- | --- | --- | --- | --- |
|  | DC | MC | MC | | |
| Zygosity: |  |  | MA | DA | Missing |
| MZ males | 595 | 1182 | 80 | 1058 | 44 |
| DZ males | 1615 | (108) | 3 | 103 | 2 |
| MZ females | 647 | 1288 | 127 | 1122 | 39 |
| DZ females | 1515 | (98) | 3 | 91 | 4 |
| DZ MF | 958 | (31) | 0 | 31 | 0 |
| DZ FM | 903 | (27) | 1 | 26 | 0 |

We employ multinomial processing tree models to analyze these data (see Multinomial processing models of source monitoring. Batchelder, W.H. & Riefer, D. M. Psychological Review, 97(4), 1990, 548-564). Figures S-1 and S-2 depict the proposed models in the MZ and DZ samples (the data of the males and females were pooled; the input for the analyses were the MZ counts, same-sex DZ counts and opposite- sex DZ counts).

*Figure S-1: Multinomial processing tree in MZ pairs.*

*Figure S-2: Multinomial processing tree in DZ pairs.*

The interpretation of the parameters is given in Table S-2.

*Table S-2 interpretation of parameters.*

| parameter | interpretation |
| --- | --- |
| a | probability of DC classification given DC is true |
| 1-g | probability of MC classification given MC is true |
| e | probability that MZ pair is DC |
| c | probability of DA given MC in MZ and DZ pairs |

The models depicted in Figures S-1 and S-2 give rise to the following probabilities of DCDA, MCMA and MCDA in the MZ and DZ pairs

MZ prob DCDA e*a + (1-e)*g

prob MCMA e*(1-a)*(1-c) + (1-e)*(1-g)*(1-c)

prob MCDA e*(1-a)*c + (1-e)*(1-g)*c

DZ prob DCDA a

prob MCMA (1-a)*(1-c)

prob MCDA (1-a)*c

In fitting these models, we have to take into account misclassification of zygosity. The probability of (DZ|MZ) (MZ misclassified as DZ) is about .03, and so prob(MZ|MZ)=.97. The probability of (MZ|DZ) (DZ misclassified as MZ) is about .10, and so prob(DZ|DZ)=.90. However, about 37% of the DZ pairs are opposite sex (i.e., prob(MZ|DZ) is zero), so that in the total DZ sample prob(MZ|DZ)=.37*0+.63*.10 = ~.063, and prob(DZ|DZ) is 1-.063=.937. Given the prior probabilities prob(MZ)=1/3 and prob(DZ)=2/3 (as observed in the NTR; consistent with the population of the Netherlands), we calculated the posterior probabilities of correct classification by means of Bayes' theorem. These are

prob(true MZ| classified MZ) =

prob(MZ|MZ)*prob(MZ) / { prob(MZ|MZ)*prob(MZ)+ prob(MZ|DZ)*prob(DZ)} =

(.97*(1/3)) / (.97*(1/3) + .063*(2/3)) = ~.89

prob(true DZ | classified DZ) =

prob(DZ|DZ)*prob(DZ) / { prob(DZ|DZ)*prob(DZ)+ prob(DZ|MZ)*prob(MZ)} =

(.937*(2/3)) / (.937*(2/3) + .03*(1/3)) = ~.98

We arrive at the probabilities below in the MZ, DZ same-sex (DZSS) and DZ opposite-sex (DZOS) samples:

MZ prob DCDA = .89*(e*a + (1-e)*g) + .11*a

prob MCMA = .89*(e*(1-a)*(1-c) + (1-e)*(1-g)*(1-c)) + .11* (1-a)*(1-c)

prob MCDA = .89*(e*(1-a)*c + (1-e)*(1-g)*c) + .11*(1-a)*c

DZSS prob DCDA = .98*a + .02*(e*a + (1-e)*g)

prob MCMA = .98*(1-a)*(1-c)) + .02*(e*(1-a)*(1-c) + (1-e)*(1-g)*(1-c))

prob MCDA = .98*(1-a)*c) + .02*(e*(1-a)*c + (1-e)*(1-g)*c)

DZOS prob DCDA = a

prob MCMA = (1-a)*(1-c))

prob MCDA = (1-a)*c)

Note that in this model the parameter g is not identified. We set this parameter to equal .05. We fitted this model to the data of the MZ, DZ same sex and DZ opposite sex twins (see below R script 1) by maximum likelihood estimation. The maximum likelihood parameter estimates are a=.9598 (s.e. = .002), c=.9413 (s.e. = .003), and e=.1408 (s.e. = .006). Based on these estimates, we have the following table of observed and expected counts (Table S-3).

*Table S-3: Observed and expected counts.*

|  |  | DCDA | MCMA | MCDA |
| --- | --- | --- | --- | --- |
| DZOS | Obs | 1861 | 1 | 57 |
|  | Exp | 1841.8 | 4.5 | 72.6 |
| DZSS | Obs | 3130 | 6 | 194 |
|  | Exp | 3155.1 | 10.2 | 164.6 |
| MZ | Obs | 1242 | 207 | 3180 |
|  | Exp | 1240.2 | 198.9 | 3189.7 |

We derived the following probabilities in the MZ pairs from the ML parameter estimates:

prob(DCDA) = .178, prob(MCMA)=.048 and prob(MCDA) = .773. Pooling with respect to amnionicity, yields the probabilities prob(DC)=.178 and prob(MC)=.773+.048 = .821. Based on these results, we constructed the processing tree as depicted in Figure S-3 to obtain the probabilities of MC and DC classification in the MZ sample.

*Figure S-3: Derived processing tree.*

Based on this processing tree, we can evaluate the effect of the zygosity misclassification and chorionicity misclassification (R script 2). Specifically we choose true values of the DCDZ, DCMZ and MCMZ correlations and then calculate the expected values based on Figure S-3. Given these probabilities we considered true DCDZ, DCMZ, and MCMZ correlations and the bias in the DCMZ and MCMZ correlations caused by these sources of misclassification. The results are shown in Tables S-4 and S-5.

*Table S-4: true and expected phenotypic correlations under misclassification. The expected correlations are biased due to the misclassification of chorionicity and zygosity**.

| true correlations | | | | expected given misclassification | | |
| --- | --- | --- | --- | --- | --- | --- |
| rDCDZ | rDCMZ | rMCMZ | diff | DCMZ | MCMZ | diff |
| .20 | .40 | .45 | .05 | .374 | .436 | .061 |
| .30 | .40 | .45 | .05 | .387 | .438 | .051 |
| .30 | .60 | .65 | .05 | .561 | .634 | .072 |
| .45 | .60 | .65 | .05 | .581 | .637 | .056 |
| .35 | .70 | .75 | .05 | .655 | .732 | .077 |
| .50 | .70 | .75 | .05 | .674 | .736 | .061 |

* *prob(DZ|MZ)=1-.97, prob(MZ|DZ)=1-.937, prob(MC|DC)=.04, and prob(DC|MC)=.05 (fixed).*

*Table S-5: true and expected phenotypic correlations under misclassification The expected correlations are biased due to the misclassification of chorionicity and zygosity**.

| true correlations | | | | expected given misclassification | | |
| --- | --- | --- | --- | --- | --- | --- |
| rDCDZ | rDCMZ | rMCMZ | diff | DCMZ | MCMZ | diff |
| .20 | .40 | .40 | .0 | .374 | .395 | .021 |
| .30 | .40 | .40 | .0 | .387 | .397 | .011 |
| .30 | .60 | .60 | .0 | .561 | .593 | .032 |
| .45 | .60 | .60 | .0 | .581 | .596 | .015 |
| .35 | .70 | .70 | .0 | .654 | .691 | .036 |
| .50 | .70 | .70 | .0 | .674 | .695 | .021 |

* *prob(DZ|MZ)=1-.97, prob(MZ|DZ)=1-.937, prob(MC|DC)=.04, and prob(DC|MC)=.05 (fixed).*

For instance, given the correlations .20, .40 and .45 (i.e., rDCDZ, rDCMZ, and rMCMZ, respectively), we obtained the expected DCMZ and MCMZ correlations as follows. Note that the probabilities of an MC classification is .1505 + .0292 + .0044 = ~.1841. The expected phenotypic correlation rMCMZ is a weighted sum of the true rMCMZ, rMCMZ and rDCDZ correlations:

expected rMCMZ: .1505/.1841*rMCMZ + .0292/.1841*rDCMZ + .044/.1841*rDCDZ = ~.395.

Similarly the expected rDCMZ is based on the the probability of DC classification, i.e., .00792 + .7014 + .1056 = ~.8149. The expected phenotypic correlation rDCMZ is a weighted sum of the true rMCMZ, rMCMZ and rDCDZ correlations:

expected rMCMZ: .00792/.8149*rMCMZ + .7014/.8149*rDCMZ + .1056/.8149*rDCDZ= ~.374.

Based on the results shown in Table S-4, we note that the two sources of misclassification result in an upwards bias in the difference in the DCMZ and MCMZ correlations. Given the range of true correlations considered, we find that the expected DCMZ vs. MCMZ difference in correlation ranges from about .051 to .077 (true difference: .05), and from .011 to .036 (true difference .0). The upward bias - regardless of its magnitude - seems not to have resulted in false positives in our results as we observe relatively few significant differences in MCMZ vs. DCMZ correlations.

# R SCRIPT 1 ..... R SCRIPT 1 ..... R SCRIPT 1

rm(list=ls(all=TRUE))

#

fitf=function(pars,xpars,ipars,MZSS,DZSS,DZOS,ipr) {

#

a=xpars[1]

c=xpars[2]

e=xpars[3]

# g=xpars[4]

g=.05 # fixed

#

if (ipars[1]) a=pars[1]

if (ipars[2]) c=pars[2]

if (ipars[3]) e=pars[3]

# if (ipars[4]) g=pars[4]

#

pEDZ=c(

a, # DCDA

(1-a)*(1-c), # MCMA

(1-a)*c) # MCDA

pEMZ=c(

e*a+g*(1-e),

e*(1-a)*(1-c)+(1-e)*(1-g)*(1-c),

e*(1-a)*c+(1-e)*(1-g)*c)

#

pmz=(1/3) # prior

pdz=1-pmz # prior

pmzmz=.97 # prob(MZ|MZ)

pdzmz=1-pmzmz # prob(DZ|MZ)

pdzdz=.937 # prob(DZ|DZ)

pmzdz=1-pdzdz # prob(MZ|DZ)

# bayes

epmz=(pmzmz*pmz)/(pmzmz*pmz+pmzdz*pdz)

epdz=(pdzdz*pdz) /(pdzdz*pdz+pdzmz*pmz)

#

pEDZSS=(epdz)*pEDZ+(1-epdz)*pEMZ #

pEMZSS=(epmz)*pEMZ+(1-epmz)*pEDZ #

# expected counts

EDZOS=pEDZ*DZOS[4]

EDZSS=pEDZSS*DZSS[4]

EMZSS=pEMZSS*MZSS[4]

logl=rep(0,3)

for (i in 1:3) {

logl[1]=logl[1]+DZOS[i]*log(DZOS[i]/EDZOS[i])

logl[2]=logl[2]+DZSS[i]*log(DZSS[i]/EDZSS[i])

logl[3]=logl[3]+MZSS[i]*log(MZSS[i]/EMZSS[i])

}

#

if (ipr) {

print("DZOS")

print(DZOS[1:3])

print(EDZOS)

#

print("DZSS")

print(DZSS[1:3])

print(EDZSS)

#

print("MZSS")

print(MZSS[1:3])

print(EMZSS)

#

print(pEMZ)

print(pEMZSS)

print(pEDZ)

print(pEDZSS)

}

logl3=2*sum(logl)

}

#

# DCDA MCMA MCDA

MZSS=c(1242,207,3180,0) # MZ

DZSS=c(3130,6,194,0) # DZSS

DZOS=c(1861,1,57,0) # DZOPP

#

MZSS[4]=sum(MZSS[1:3]) # total

DZSS[4]=sum(DZSS[1:3]) # total

DZOS[4]=sum(DZOS[1:3]) # total

# a c e

pars=c(.9,.9,.3) # starting values

xpars=pars

lower=c(.001,.001,.001) # low bounds

upper=c(.999,.999,.999) # upper bounds

ipars=c(T,T,T) # T=free parameter

ipr=F # print results

go=T # fit the model

if (go) {

res1=optim(pars,fitf,method ="L-BFGS-B",lower=lower,upper=upper,hessian=TRUE,

xpars=xpars,ipars=ipars,MZSS=MZSS,DZSS=DZSS,DZOS=DZOS,ipr=ipr)

ipr=T

test=fitf(res1$par,xpars,ipars,MZSS,DZSS,DZOS,ipr)

se=sqrt(diag(solve(res1$hessian)))

}

# R SCRIPT 2 ..... R SCRIPT 2 ..... R SCRIPT 2

rm(list=ls(all=TRUE))

rMZMC=.70

rMZDC=.70

rDZDC=.50

#

# mc dc mc dc mc dc

p=c(.89*.178*.95, .89*.178*.05, .89*.821*.04, .89*.821*.96, .11*.04, .11*.96)

pmc= (p[1]+p[3]+p[5])

pdc= (p[2]+p[4]+p[6])

obsrMZMC = (p[1]/pmc)*rMZMC + (p[3]/pmc)*rMZDC + (p[5]/pmc)*rDZDC

obsrMZDC = (p[2]/pdc)*rMZMC + (p[4]/pdc)*rMZDC +(p[6]/pdc)*rDZDC

print(c(rMZMC, rMZDC, obsrMZMC, obsrMZDC, obsrMZMC-obsrMZDC))
